# Xtr-mediated regulation of 4Fe-4S binding protein gene confers resistance to oxidative and antibiotic stress in *Clostridioides difficile*

**DOI:** 10.3389/fmicb.2026.1817884

**Published:** 2026-05-29

**Authors:** Wenjing Wu, Jia Zhou, Fahui Chen, Shan Liu, Yuan Xie, Guzhen Cui, Zhizhong Guan, Yan Zhao, Wei Hong

**Affiliations:** 1 Key Laboratory of Endemic and Ethnic Diseases, Ministry of Education, Guizhou Medical University, Guiyang, Guizhou, China; 2 Collaborative Innovation Center for Prevention and Control of Endemic and Ethnic Regional Diseases Co-constructed by the Province and Ministry, Guiyang, Guizhou, China; 3 Key Laboratory of Microbiology and Parasitology of the Education Department of Guizhou, Guizhou Medical University, Guiyang, China

**Keywords:** 4Fe-4S binding protein, *Clostridioides difficile*, gene expression regulation, stress resistance, xtr

## Abstract

**Background:**

Xenobiotic response element (XRE) family transcriptional regulators play crucial roles in diverse bacterial physiological processes and environmental adaptation. However, the functional characterization of these regulators in *Clostridioides difficile* remains unclear.

**Methods:**

In this study, we employed the Allele-coupled exchange (ACE) gene knockout technique to construct knockout (Δ*xtr*) and complemented (::*xtr*) mutants of the *xtr* gene, which encodes a member of the XRE family protein in *C. difficile*, and systematically analyzed the resulting phenotypic alterations. Subsequently, transcriptome sequencing, EMSA, and reporter gene assays were utilized to investigate how *xtr* regulates 4Fe-4S binding protein gene (*4Fe-4S_BP*, *CD17960*).

**Results:**

The Δ*xtr* mutant exhibited a statistically significant reduction in hydrogen peroxide tolerance, as well as decreased susceptibility to vancomycin and metronidazole. Concomitantly, the expression of virulence-related genes and cellular toxicity, as well as growth rate and autolysis, were all notably diminished. Transcriptomic analysis revealed a significant downregulation of *4Fe-4S_BP* in the Δ*xtr* mutant (3.69-fold). Overexpression of the *4Fe-4S_BP* in the Δ*xtr* background restored hydrogen peroxide tolerance to wild-type levels. Electrophoretic Mobility Shift Assays (EMSA) confirmed that the recombinant Xtr protein directly binds to the promoter region of *4Fe-4S_BP*. Reporter gene assays utilizing *gusA* demonstrated that Xtr activates transcription from the *4Fe-4S_BP* promoters.

**Conclusion:**

These findings elucidate a molecular mechanism by which Xtr positively regulates the expression of *4Fe-4S_BP*, thereby modulating *C. difficile* tolerance to hydrogen peroxide, metronidazole, and vancomycin. This work provides novel experimental evidence for a deeper understanding of the environmental adaptation strategies and virulence regulatory networks employed by *C. difficile*.

## Introduction

*Clostridioides difficile* (*C. difficile*) is a strictly anaerobic, spore-forming bacterium and the primary pathogen responsible for antibiotic-associated diarrhea. It is considered the most common cause of hospital-acquired diarrhea and poses a significant threat to global healthcare systems, with approximately 500,000 infections and over 29,000 deaths annually (Chandrasekaran and Lacy, 2017). The economic burden of *C. difficile* infection (CDI) on U.S. hospitals is estimated at $496 million annually. The virulence of *C. difficile* is primarily determined by toxins A and B. These toxins inactivate Rho-GTPases, which are crucial regulators of the actin cytoskeleton, through a process of glycosylation. This inactivation leads to cytoskeletal disruption and death of intestinal epithelial cells (Fettucciari et al., 2017). Inflammatory and immune cells release numerous mediators, causing a large influx of neutrophils, which ultimately results in diarrhea, intestinal bleeding, perforation, and toxic megacolon (Solomon, 2013).

Oxidative stress plays a crucial role in microbe-host interactions. Maintaining a vegetative cellular state in the face of oxidative stress is a prerequisite for pathogenic microorganisms to establish infection. During CDI, bacteria encounter reactive oxygen species (ROS) – including superoxide anion (O_2_^∙−^), hydrogen peroxide (H_2_O_2_), hydroxyl radicals (∙OH), and peroxynitrite (ONOO^–^), et al. – generated by human intestinal neutrophils, macrophages, and antibiotics (Kohanski et al., 2007; Li et al., 2015). Certain antibiotics also utilize ROS to inhibit bacterial growth (Dwyer et al., 2009); for example, under anaerobic conditions, bacterial electron transport chains reductively activate metronidazole, progressively converting its nitro group (-NO_2_) to unstable intermediates like nitro anion radicals (R-NO_2_^–^). These intermediates further decompose, generating highly reactive hydroxyl radicals (∙OH). These ROS species can damage microbial DNA, proteins, and lipids, leading to bacterial cell death (Rodriguez Ferreiro et al., 2002). It has been demonstrated that the Fur family regulator PerR and the alternative sigma factor σ*^B^* mediate the oxidative stress response in *C. difficile* (Kint et al., 2017; Troitzsch et al., 2021). Iron-sulfur clusters, comprised of Fe^2+^ and S^2–^, are among the oldest known cofactors, widely distributed in both prokaryotic and eukaryotic cells. In 4Fe-4S dehydratases, three iron atoms within the cluster are coordinated to the protein scaffold via cysteine residues, while the fourth iron atom is solvent-exposed and functions as a Lewis acid, coordinating with the substrate (Bandyopadhyay et al., 2008). ROS can oxidize the exposed iron atom within [4Fe-4S] clusters, leading to cluster degradation, release of catalytic iron, and subsequent neutralization of excess ROS (Anand et al., 2021). In bacteria such as *Escherichia coli* (*E. coli*), the unique 4Fe-4S cluster in fumarase inhibits the irreversible oxidative degradation of the cluster by hydrogen peroxide, protecting enzyme activity and helping the bacteria resist host-immune-mediated oxidative stress (Lu and Imlay, 2019). Proteins that harbor a 4Fe-4S cluster utilize it as an inherent catalytic or structural cofactor, whereas 4Fe-4S binding proteins specifically refer to a class of chaperones or carrier proteins involved in the synthesis, repair, or sequestration of iron–sulfur clusters. The two categories differ in their functional roles and physiological significance.

Pathogenic microorganisms have evolved mechanisms to resist oxidative stress during long-term co-evolution with their hosts. Xenobiotic response element (XRE) family redox-sensing transcriptional regulators [The main features of XRE regulators are a conserved N-terminal helix-turn-helix (HTH) DNA-binding domain, through which they recognize and bind to XRE elements in the promoter regions of target genes, thereby participating in the regulatory response to xenobiotic substances (such as antibiotics and toxins). In addition, some family members also possess a C-terminal ligand-binding domain for sensing small molecule signals] were first identified in *Streptomyces coelicolor* (Richardson et al., 2015) and subsequently found to be broadly distributed in both eukaryotic and prokaryotic organisms (Trouillon et al., 2021; Zhang et al., 2022). Their N-terminal DNA-binding domain, containing a helix-turn-helix motif, binds to the promoter regions of target genes to regulate transcription. The structural diversity of the C-terminal region confers a wide range of functionalities. In *Streptococcus suis*, XRE family transcriptional regulators SrtR and XtrSs play critical roles in resisting host oxidative stress (Hu et al., 2019; Long et al., 2022; Zhu et al., 2018). Hu et al. reported that the *SrtR* protein regulates *Streptococcus suis* resistance to oxidants and high temperatures (Hu et al., 2019). Xiang Long et al. demonstrated that the XRE family transcriptional regulator LfsT enhances metabolic activity in *Pseudomonas aeruginosa*, while reducing host resistance to phages (Long et al., 2022). In *Brucella abortus*, the HTH-Xre toxin-antitoxin system mediates resistance to hydrogen peroxide-induced oxidative stress by regulating the expression of antioxidant enzyme genes, including *katE*, *ahpC*, and *oxyR* (Gómez et al., 2023). In *Corynebacterium glutamicum*, MsrR, an XRE family regulator involved in oxidative stress sensing, dissociates from promoter regions under oxidative conditions through a Cys62-based oxidation-sensing mechanism, thereby modulating the expression of the downstream msrR-3-mst and *mfs* genes (Si et al., 2020). Thus, the functions of XRE family transcriptional regulators are diverse. Nevertheless, our understanding of how XRE family transcriptional regulators regulate bacterial antioxidant capacity in *C. difficile* remains limited.

In this study, we identified an ortholog of Redox-sensing transcriptional repressor (designated *xtr*) in *C. difficile* and successfully constructed an *xtr* deletion mutant using ACE methodology (Heap et al., 2012). By comparing the wild-type strain (WT) with the Δ*xtr* mutant, we elucidated the regulatory role of Δ*xtr* in peroxide stress, antibiotic resistance, and toxin gene expression. The Δ*xtr* mutant exhibited decreased tolerance to H_2_O_2_ and multiple antibiotics, with significantly reduced expression of the toxin genes *tcdA* and *tcdB*, diminished cytotoxicity, and accelerated autolysis. Transcriptomic data analysis further revealed that the *xtr* gene affects the expression of 4Fe-4S binding protein gene (*4Fe-4S_BP*, *CD17960*). Most importantly, we demonstrated that the mRNA level of *xtr* is upregulated upon exposure to oxidative stress and that Xtr exerts a global regulatory role on oxidative stress resistance, antibiotic resistance, toxin production, and autolysis in *C. difficile* (Chen et al., 2009). These findings expand our understanding of the function of XRE family transcriptional regulators in the important pathogen *C. difficile* and provide beneficial insights into *C. difficile* adaptation to the intestinal environment.

## Materials and methods

### Strain and culture conditions

The strains used in this study are listed in Supplementary Table S1. The *Escherichia coli* (*E. coli*) NE Express strain (C2523I, New England Biolabs, Ipswich, United States of America) was utilized as the competent cell for standard cloning experiments and for constructing gene editing plasmids. *E. coli* strain CA434 served as the donor for conjugative transformation plasmids (Purdy et al., 2002). *C. difficile* 630 strain (WT) was cultured in BHIS medium (BHIS medium preparation: Dissolve 38.5 g/L brain heart infusion powder, 1 g/L L-cysteine, and 45 g/L yeast extract in 1 L of ddH_2_O, then sterilize by autoclaving. To prepare solid medium, add 1.5% (w/v) agar powder as needed) (Zhang et al., 2020). The *E. coli* strains were grown in Luria-Bertani (LB) medium. ampicillin (15 μg/mL), cefotaxime (8 μg/mL), d-cycloserine (250 μg/mL), or kanamycin (250 μg/mL) were added to the BHIS or LB medium when appropriate. 1.5% Agar powder (9002-18-0, Solarbio, Beijing, China) was used to prepare solid LB and BHIS media for culturing *E. coli* and *C. difficile* (w/v = 1.5 g/mL). 5-fluoroorotic acid (5-FOA) was dissolved in dimethyl sulfoxide (DMSO) at 100 mg/mL storage concentration, with a 2 mg/mL working concentration in the BHIS medium (*C. difficile* minimal medium, CDMM) CDMM was used for gene recombination screening (Ehsaan et al., 2016).

### Plasmids construction

Plasmids and primers used in the present study are listed in Supplementary Tables S1, S2. The molecular cloning was performed using restriction enzymes purchased from New England Biolabs (Beijing). The DNA polymerase 2 × Phanta Flash Master Mix (Dye Plus, #P520, Vazyme Biotech Co., Ltd, Nanjing, China) was employed to amplify gene fragments. The construction of the *pyrF* gene knockout plasmid is based on our previous work (Yang et al., 2021) and is briefly described as follows: Primers WH731| WH732 and WH733| WH734 pairs were used to amplify the upstream (798 bp) and downstream (765 bp) homologous arms of the *pyrF* gene. The upstream and downstream arms were then used as templates, and primers WH731| WH734 were employed to join the homologous arms. The ClonExpress Ultra One Step Cloning Kit (C115-02, RRID, Vazyme Biotech Co., Ltd., Nanjing, China) was used for seamless cloning, integrating the connected DNA fragment into the *Hind* III site of the pMTL82151 plasmid. Primers WH681| WH682 were employed to screen for the correct DNA recombinant plasmid, verified by sequencing (PCR products were sequenced by Shanghai Sangon Biotech Co., Ltd. using the Sanger dideoxy sequencing method), and named pJJB1.

The process of construction of gene knockout plasmid pJJB-DE1 for *xtr* is noted as follows: primer pair HW601| HW602 was used to amplify the *pyrF* gene expression cassette from the genome of *Clostridium beijerinckii* 8052 (Wang et al., 2013). The PCR product obtained was then assembled into the *Pme* I site of the plasmid pMTL82151 using the homologous recombination cloning method (Xia et al., 2019), generating the plasmid pJJB-DE. Primer pairs HW621| HW622 and HW623| HW624 were used to amplify the upstream (1,351 bp) and downstream (567 bp) arms of the *xtr* gene, respectively. Most of the homologous arms are located within the *xtr* gene, and recombination with the *xtr* genome will result in the deletion of 152 bp from this gene (740 bp vs. 588 bp). The ClonExpress Ultra One Step Cloning Kit (C115-02, RRID, Vazyme Biotech Co., Ltd, Nanjing, China) was utilized to seamlessly assemble the PCR products with *Hind* III-linearized pJJB-DE plasmid, resulting in the *xtr* gene knockout plasmid pJJB-DE1.

Primers WH731| WH734 (Supplementary Table S2) were utilized to amplify the *pyrF* expression cassette from the *C. difficile* CD630 genome to construct the gene complementation plasmids. The PCR products were assembled with *Hind* III-linearized pMTL82151 to obtain the *pyrF* gene complementation vector, pJJBC1 (Supplementary Table S1). Similarly, primers HW671| HW674 (Supplementary Table S2) were used to amplify the *xtr* gene expression cassette from the *C. difficile* CD630 genome. The PCR products were assembled with *Hind* III-linearized pMTL82151 plasmid to generate the *xtr* gene complementation plasmid, designated as pJJBC2 (Supplementary Table S1).

### Construction of Δ*pyrF* strain

The knockout plasmid pJJB1 was constructed by amplifying the upstream and downstream homology arms of the *pyrF* gene via fusion PCR and seamlessly cloning the fused fragment into the *Hind* III site of the pMTL82151 plasmid. This pJJB1 plasmid was introduced into the recipient strain *C. difficile* CD630 from the donor strain *E. coli* CA434 via conjugation. Thiamphenicol-resistant (Tm) exconjugants were first selected on BHIS-Tm agar plates, and then these Tm^r^ colonies were streaked onto BHIS-FOA solid medium containing 400 μg/mL 5-fluoroorotic acid (5-FOA) and incubated for 24–48 h. Because the wild-type *pyrF* gene converts 5-FOA into the toxic compound 5-fluorouracil, only strains that had undergone double-crossover homologous recombination resulting in deletion of the endogenous *pyrF* gene were able to survive. Candidate Δ*pyrF* mutants were screened by PCR using primers WH735/WH736 (Supplementary Table S2), with the mutant strain producing a shorter amplicon (1,598 bp) compared to the WT (2,014 bp). Finally, the confirmed Δ*pyrF* mutant was consecutively passaged several times in BHIS medium to cure the plasmid pJJB1 (Hong et al., 2018).

### Construction of the Δ*xtr* mutant

To construct the Δ*pyrF*Δ*xtr* mutant strain, the pBJJ-DE1 plasmid was transferred into the Δ*pyrF* mutant strain. The resulting transformants were spread on CDMM selective medium and incubated anaerobically for approximately 72 h. The formed transformants were screened for single-crossover mutants using HW681| HW636 and HW635| HW636 primers (Supplementary Table S2). The single-crossover strains were streaked on CDMM medium supplemented with 2 mg/mL 5-FOA and 20 μmol/mL uracil, with the HW635| HW639 (Supplementary Table S2) primers employed to select the Δ*pyrF*Δ*xtr* double-crossover mutants.

To obtain the Δ*xtr* mutant strain, the pBJJ-C1 plasmid was transferred into the Δ*pyrF* mutant. Tm-resistant transformants were spread on CDMM medium and cultured anaerobically at 37 °C for 24–48 h. Colonies formed on the plate were screened using HW735| HW736 (Supplementary Table S2) primers to identify the *pyrF* gene complementation (::*pyrF*, recombination in the genome) in the Δ*pyrF*Δ*xtr* mutant strain. The pBJJ-C2 plasmid was used for *xtr* gene over-expression to obtain the ::*xtr* complementation strain.

### Sequence alignment

The amino acid sequence for *CD04490* (*xtr*) was initially retrieved from the National Center for Biotechnology Information (NCBI) database.^1^ Subsequently, Protein BLAST^2^ was employed using the RefSeq_select reference database to identify homologous protein sequences from *C. difficile* and other representative strains. FASTA files of these sequences were downloaded for further analysis. The collected homologous sequences were then submitted to the CLUSTALW multiple sequence alignment server.^3^ Global alignment was performed using default parameters, generating a standard.aln alignment file. The resulting alignment was visualized and refined using Jalview software. Conservation levels were indicated by asterisks (*) for fully conserved residues, colons (:) for highly similar residues, and periods (.) for generally similar residues. The editable alignment was exported as a scalable vector graphic (SVG) file. To predict the transmembrane structure of the target protein, the amino acid sequence was analyzed using the TMHMM 2.0 server.^4^ Potential transmembrane helical regions were identified. Finally, a composite graphic was generated depicting transmembrane domains (TM), conserved residues (indicated by asterisks/black shading), and levels of similarity (indicated by asterisks/colons/gray shading).

### RT-qPCR

Genomic DNA was removed, and total RNA was extracted from WT, Δ*xtr*, and ::*xtr* strains using the Bacterial Total RNA Extraction Kit following the manufacturer’s protocol (DP430, Tiangen, Beijing, China). The FasKing gDNA Dispelling RT SuperMix (KR118, Tiangen, Beijing, China) was used to reverse-transcribe the extracted total RNA into cDNA. The expression level of the *xtr* gene (HW691| 692) (Supplementary Table S2) in different mutant strains was analyzed using the 16S rRNA gene as the reference (HW554| HW555), and histograms were generated using Prism 6.0 software (v.10.1.2(324), GraphPad Software, Inc, San Diego, United States).

The RNA extraction procedures for Δ*xtr*::*4Fe-4S_BP* were performed as described above. Using the 16S rRNA gene (HW554| HW555) (Supplementary Table S2) as a reference, the expression levels of the *4Fe-4S_BP* (HW2602| 2603) (Supplementary Table S2) in different strains were analyzed, and histograms were generated using Prism 10.0 software (v.10.2.0).

### Growth curve

The WT, Δ*xtr*, and ::*xtr* strains were inoculated at 1% (v/v) into 5 mL of BHIS medium. Subsequently, they were anaerobically cultured at 37 °C, and the OD_600_ values were measured every 2 h using a spectrophotometer (Ultrospec 10, Amersham Biosciences, GE) for a duration of 28 h. The growth curves were plotted using Prism 6.0 software (v.10.1.2(324), GraphPad Software, Inc., San Diego, United States).

### Autolysis rate assay

Following the method described by El Meouche et al. (2013). the autolysis rates of the WT, Δ*xtr*, and ::*xtr* were analyzed. The WT, Δ*xtr*, and ::*xtr* strains were cultured in BHIS medium until the OD_600_ reached 0.6 and 0.8. The bacterial broth was centrifuged at 1,530 × g for 5 min, the supernatant was discarded, and the bacterial pellet was washed twice with PBS (Phosphate-Buffered Saline, PBS) and resuspended in 50 mM potassium PBS buffer (C10010500BT, Gibco, New York, United States) containing 0.01% Triton X-100. The cultures were incubated under anaerobic conditions at 37 °C, and OD_600_ absorbance values were recorded at 20-min intervals. The initial OD_600_ value was defined as 0% autolysis, with three biological replicates conducted for each experiment.

### Scanning electron microscope (SEM)

Single colonies of the WT, Δ*xtr*, and ::*xtr* were individually picked and cultured in BHIS medium until the optical density at 600 nm (OD_600_) reached 0.6. Subsequently, the bacterial suspension was centrifuged at 4,000 × g for 3 min to harvest the cell pellets. The pellets were resuspended in 2.5% glutaraldehyde and fixed overnight at 4 °C. After fixation, the bacterial cells were washed three times with PBS, followed by gradient dehydration in 50, 70, 90, and 100% ethanol for 5 min per concentration. The dehydrated samples were dried in a vacuum freeze dryer. A small amount of the dried bacterial powder was gently picked up with a sterile toothpick, adhered to a conductive carbon tape, and sputter-coated with gold using a vacuum coater to enhance electrical conductivity and imaging quality. Finally, the tape with immobilized samples was mounted on a glass slide, and the surface morphology of the strains was observed using a scanning electron microscope (SEM, model S-3400, Hitachi, Tokyo, Japan) (Yang et al., 2025).

### Western blot

To further clarify the differences in TcdA and TcdB levels among the various mutant strains, Western blot analysis was performed to detect the expression of TcdA and TcdB in WT, Δ*xtr*, and ::*xtr* strains. The experimental steps are summarized as follows (Yang et al., 2025): bacterial culture was centrifuged to collect the supernatant, which was then filtered through a 0.22 μm sterile membrane. The total protein content in the supernatant was determined using the BCA method (Hu et al., 2010), and 6 μg of protein-containing supernatant was subjected to (Sodium Dodecyl Sulfate–Polyacrylamide Gel Electrophoresis, SDS-PAGE) SDS-PAGE. Subsequent steps followed the Western blot protocol as previously described (Yang et al., 2025).

### Hydrogen peroxide tolerance

To assess the tolerance of WT, Δ*xtr*, ::*xtr*, and Δ*xtr*::*4Fe-4S_BP* overexpressing strains to oxidative stress, each strain was first streaked onto solid agar plates and a single colony was selected to inoculate BHIS liquid medium. Cultures were grown anaerobically at 37°C until reaching the logarithmic growth phase (OD_600_ = 0.6).

A stock solution of 30% hydrogen peroxide (H_2_O_2_) was used to prepare a series of H_2_O_2_ working solutions in BHI medium, ranging from 0 to 100 nM in 5 nM increments. Logarithmically growing cells of each strain were then inoculated at 5% into 96-well plates containing the different concentrations of H_2_O_2_ (200 μL per well). The plates were incubated anaerobically at 37°C for 24 h, and the OD_600_ of the bacterial suspensions in each well was measured to evaluate the strains’ tolerance to oxidative stress.

### Cytotoxicity assay

Cytotoxicity assays were performed on African green monkey kidney (Vero cells are a well-established, gold-standard cell line for quantifying the cytotoxicity of *C. difficile* TcdA/TcdB, due to their extremely high sensitivity to these two specific toxins and their well-documented cytopathic rounding response, despite not being of intestinal origin (Xie et al., 2014).) cells using WT, Δ*xtr*, and *::xtr* strains. Vero cells were maintained in high-glucose Dulbecco’s modified Eagle medium (DMEM) supplemented with 10% fetal bovine serum and 1% penicillin-streptomycin solution (10 mg/mL streptomycin and 10,000 U/mL penicillin).

The experimental procedure was conducted as follows: Vero cells were seeded into 24-well plates and incubated at 37°C until a confluent monolayer formed. After aspirating the medium, cells were gently washed three times with sterile phosphate-buffered saline (PBS) and then switched to serum- and antibiotic-free high-glucose DMEM for 24 h starvation.

Meanwhile, WT, Δ*xtr*, and *::xtr* strains were cultured in BHIS medium at 37°C with shaking until the optical density at 600 nm (OD_600_) reached 1.0. Bacterial suspensions were centrifuged at 12,000 rpm for 5 min, and the resulting supernatants were collected and filtered through a 0.22 μm membrane to remove residual bacteria, yielding toxin-containing supernatants.

The toxin-containing supernatants were serially diluted from 0.5 × 10^1^ to 0.5 × 10^10^;. Then, 200 μL of each dilution was added to individual wells and incubated overnight at 37°C. Following incubation, cell morphology was observed and recorded under a light microscope at 200 × magnification. Cytotoxicity was determined based on the highest dilution that induced rounding and morphological changes in Vero cells (Yang et al., 2025).

### CCK-8 assay for cell viability

Vero cells were revitalized and cultured, then seeded into 24-well plates at a volume of 1 mL per well, followed by overnight incubation. Supernatants from the WT, Δ*xtr*, and ::*xtr* strains were collected, filtered through a 0.22-μm pore-size filter, and stored for subsequent use. The corresponding supernatants were added to each well and incubated overnight. Subsequently, 100 μL of 10% CCK-8 solution was added to each well. After 2 h of incubation, 200 μL aliquots were transferred to a 96-well plate, and the absorbance was measured at 450 nm using a microplate reader.

### Antibiotic resistance

Antibiotic susceptibility testing was performed using antibiotic-containing BHI agar plates to determine the Minimum Inhibitory Concentration (MIC). The procedure was as follows: BHI solid medium was prepared, and the MICs to metronidazole, vancomycin were determined using the agar dilution method. Logarithmically growing bacterial cultures were diluted to approximately 1 × 10^8^ CFU/mL. Each strain was spotted onto agar plates containing varying concentrations (0–256 μg/mL) of each antibiotic, with antibiotic-free plates serving as a control. Three replicates were performed for each strain. MIC values were obtained according to the guidelines outlined in “Methods for Antimicrobial Susceptibility Testing of Anaerobic Bacteria (M11-Ed9)” (2018) (CLSI, 2018), recommended by the Clinical and Laboratory Standards Institute (CLSI).

### Transcriptome analysis

RNA was extracted when the WT and Δ*xtr* strains were grown to the logarithmic phase (OD_600_ = 0.8), Total RNA was isolated using the Trizol Reagent (Invitrogen Life Technologies). Quality and integrity were determined using a NanoDrop spectrophotometer (Thermo Scientific) and a Bioanalyzer 2100 system (Agilent). Zymo-Seq RiboFree Total RNA Library Kit removed rRNA from total RNA. Random oligonucleotides and SuperScript III were used to synthesize the first strand cDNA. Second-strand cDNA synthesis was subsequently performed using DNA Polymerase I and RNase H. Remaining overhangs were converted into blunt ends via exonuclease/polymerase activities, and the enzymes were removed (Add an equal volume of AMPure XP beads to the reaction mixture, pipette to mix thoroughly, and incubate at room temperature for 5 min. Place the tube on a magnetic stand for 2–5 min until the solution becomes clear, then carefully remove the supernatant. Wash the beads with 200 μL of 80% ethanol, let stand for 30 s, and remove the ethanol; repeat this wash once. Air-dry the beads at room temperature for 3–5 min, add an appropriate volume of nuclease-free water or elution buffer, mix well, and incubate at room temperature for 2 min. Place the tube back on the magnetic stand to separate, and collect the supernatant to obtain the purified fragments). After adenylation of the 3’ ends of the DNA fragments, Illumina PE adapter oligonucleotides were ligated to prepare for hybridization. To select cDNA fragments of the preferred 400–500 bp in length, the library fragments were purified using the AMPure XP system (Beckman Coulter, Beverly, CA, United States). DNA fragments with ligated adaptor molecules on both ends were selectively enriched using Illumina PCR Primer Cocktail in a 15-cycle PCR reaction. Products were purified (AMPure XP system) and quantified using the Agilent high-sensitivity DNA assay on a Bioanalyzer 2100 system (Agilent). The sequencing library was then sequenced on NovaSeq 6000 platform (Illumina) by Shanghai Personal Biotechnology Cp. Ltd. (Song et al., 2023).

### Protein preparation and electrophoretic mobility shift assay

To construct an expression plasmid for Xtr, the full-length open reading frame of the *xtr* gene was amplified using primers HW2218 and HW2384 (Supplementary Table S2) and inserted into the pET-28a vector. The resulting recombinant plasmid (Supplementary Table S1), pET-Xtr (Supplementary Figure S1), was transformed into *E. coli* BL21. Induction and purification of the His6-tagged Xtr protein (designated His6-Xtr) were performed as described in reference (Wang et al., 2023). The purified His6-Xtr protein was quantified using the BCA assay, and its purity was verified by SDS-PAGE. Potential gene promoter regions were predicted using BPROM.^5^ To identify potential Xtr binding motifs, we selected 8 genes (Supplementary Table S3) that were differentially expressed in the Δ*xtr* mutant based on RNA-seq data and are involved in oxidative stress response-related processes. The 1,000 bp sequences upstream of the start codon of each gene were extracted from the *C. difficile* 630 genome. These 8 promoter sequences were submitted to the MEME Suite^6^ for analysis. DNA probe (The probe used for the EMSA assay was a 38 bp (5′-gtatggaccttgcatatgcaccacctttttcaacagct-3′) fluorescently labeled DNA fragment of the promoter of the *4Fe-4S_BP*) preparation and the Electrophoretic Mobility Shift Assay (EMSA) were conducted according to the methods described in reference (Wang et al., 2023).

### Xtr-activated promoter rescue assay

A 500 bp region upstream of the *4Fe-4S_BP* gene, containing the core binding sequence (5’-GCATATGCACCACC-3’) of the promoter fragment, was amplified from *C. difficile* CD630 genomic DNA using primers HW2620 and HW2621 (Supplementary Table S2). The resulting amplicon was digested with *Bam*H I restriction enzyme (NEB, Beijing) and ligated into the pMTL82151-*gusA* vector to construct the recombinant reporter plasmids pWWJ3 and pWWJ4 (Supplementary Figure S2 and Supplementary Table S1). These plasmids were transformed into *E. coli* BL21.

Taking advantage of the ability of β-glucuronidase (GUS) to catalyze the substrate X-Gluc, generating a blue precipitate, and to hydrolyze 4-methylumbelliferyl-β-D-glucuronide (4-MUG, Source BioScience) producing the fluorescent product 4-methylumbelliferone (4-MU) and β-D-glucuronide, both qualitative staining and quantitative fluorescence assays were used to assess the transcriptional activity of the GUS enzyme activity, which indicates the activity of the target promoter (Perry et al., 2006).

### Data analysis

Statistical analyses were carried out with GraphPad Prism 10 (version 10.2.0). For comparisons involving two groups, Student’s *t*-test or appropriate non-parametric tests were used. Comparisons across multiple groups were performed using one-way ANOVA, whereas time-course comparisons among multiple groups were analyzed by two-way ANOVA followed by Tukey’s *post-hoc* test. Results are expressed as mean ± standard deviation. Statistical significance was defined as *P*<0.05 at α = 0.05 (*n* = 3). Significance levels are indicated as follows: ns (*P* > 0.1); * (*P* < 0.05); ** (*P* < 0.01); *** (*P* < 0.001); **** (*P* < 0.0001).

## Results

### Construction of the Δ*xtr* mutant and phenotypic characterization

Through protein sequence homology analysis, we identified a gene (*CD04490*, designated *xtr*) in the *C. difficile* genome that encodes a protein with high homology to Xenobiotic response element (XRE) family transcription factors in *Listeria seeligeri* (67.9%), *Listeria monocytogenes* (64.5%), *Enterococcus faecalis* (88.44%), and a *Clostridiales bacterium* (66.58%) (Supplementary Figure S3). Using Allele-Coupled Exchange (ACE) method (Ng et al., 2013), we constructed a Δ*xtr* knockout mutant and a complementation strain ::*xtr* (Supplementary Figure S4).

**FIGURE 1:**
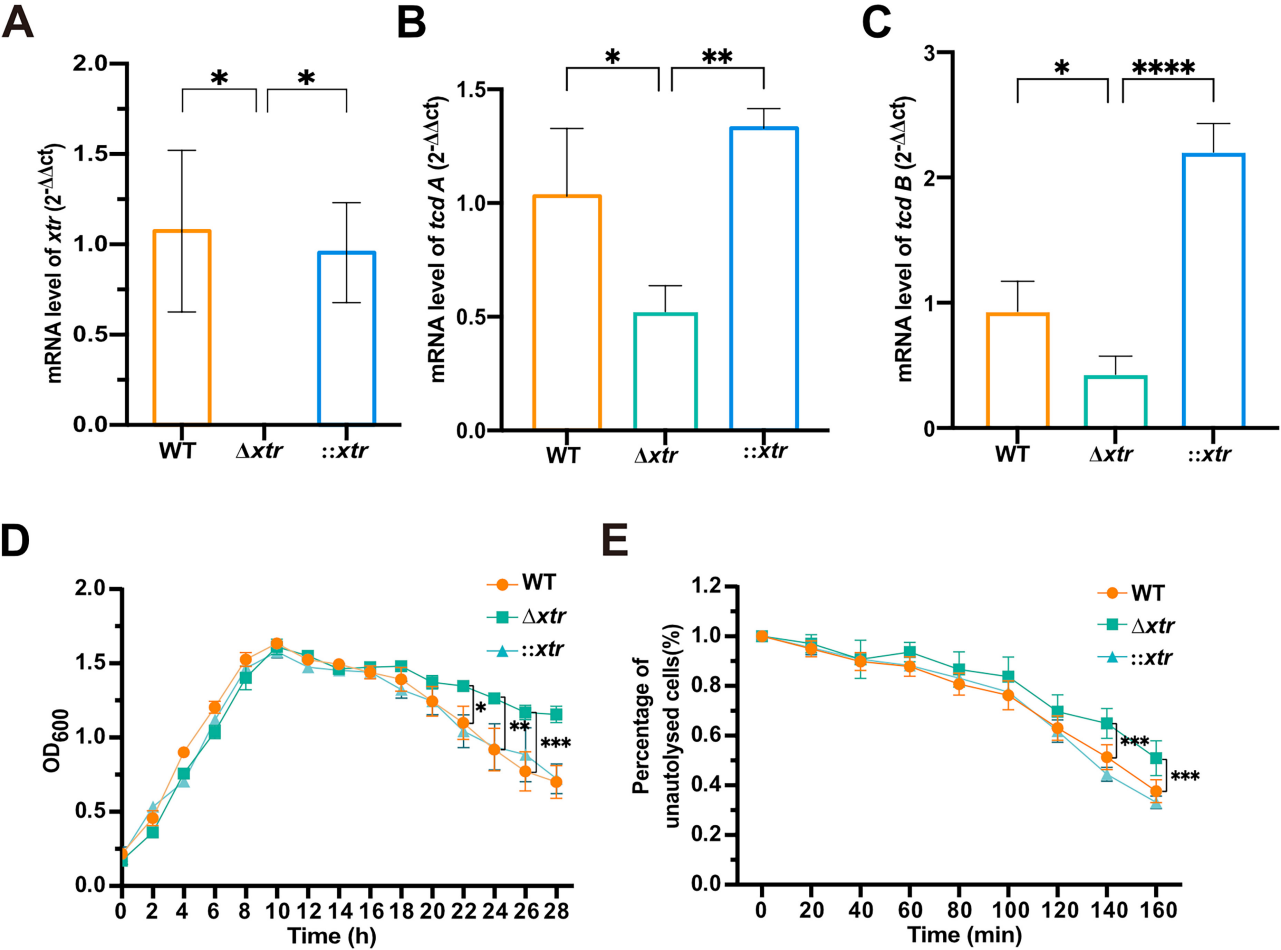
Phenotypic characteristics of the mutant strains. **(A)** RT-qPCR analysis of *xtr* expression in WT, Δ*xtr*, and ::*xtr* strains. **(B,C)** RT-qPCR analysis of *tcdA* **(B)** and *tcdB* **(C)** expression in WT, Δ*xtr*, and ::*xtr* strains. **(D)** Growth curves of WT, Δ*xtr*, and ::*xtr* strains. The x-axis represents incubation time (hours), and the y-axis represents optical density at 600 nm (OD_600_). **(E)** Autolysis curves of WT, Δ*xtr*, and ::*xtr* strains. The x-axis represents Triton X-100 treatment time, and the y-axis represents the percentage of intact cells. Data are presented as mean ± SEM, (*n* = 3). Statistical significance was determined by one-way ANOVA followed by Tukey’s *post-hoc* test. *P < 0.05, **P < 0.01, ***P < 0.001, ****P < 0.0001.

First, we compared the expression levels of the *xtr* gene in *C. difficile* WT, Δ*xtr*, and ::*xtr* strains using RT-qPCR. The results showed that *xtr* gene expression was absent in the Δ*xtr* strain, while the *xtr* expression level in the ::*xtr* strain was not significantly different from that of the WT strain (Figure 1A), confirming successful construction of the Δ*xtr* and ::*xtr* strains. We then assessed the expression levels of the toxin genes *tcdA/tcdB*, finding that transcription of both *tcdA/tcdB* was significantly reduced in the Δ*xtr* strain, while expression levels were restored in the ::*xtr s*train (Figures 1B,C).

Next, we examined the growth rate and autolysis rate of the Δ*xtr* mutant. The results demonstrated that deletion of the *xtr* gene had no significant effect on the growth rate of *C. difficile.* However, the autolysis rate of the Δ*xtr* strain was slower compared to the WT and ::*xtr* strains (Figure 1D). To further confirm this, we treated the strains with the detergent Triton X-100 to assess autolysis rates. The results showed that the autolysis rate of the Δ*xtr* strain remained significantly lower than that of the WT and ::*xtr* strains (Figure 1E). In summary, deletion of the *xtr* gene had no significant effect on bacterial growth rate, but it led to downregulation of toxin gene expression and a reduced autolysis rate in *C. difficile.*

### Changes in cell surface morphology

**FIGURE 2:**
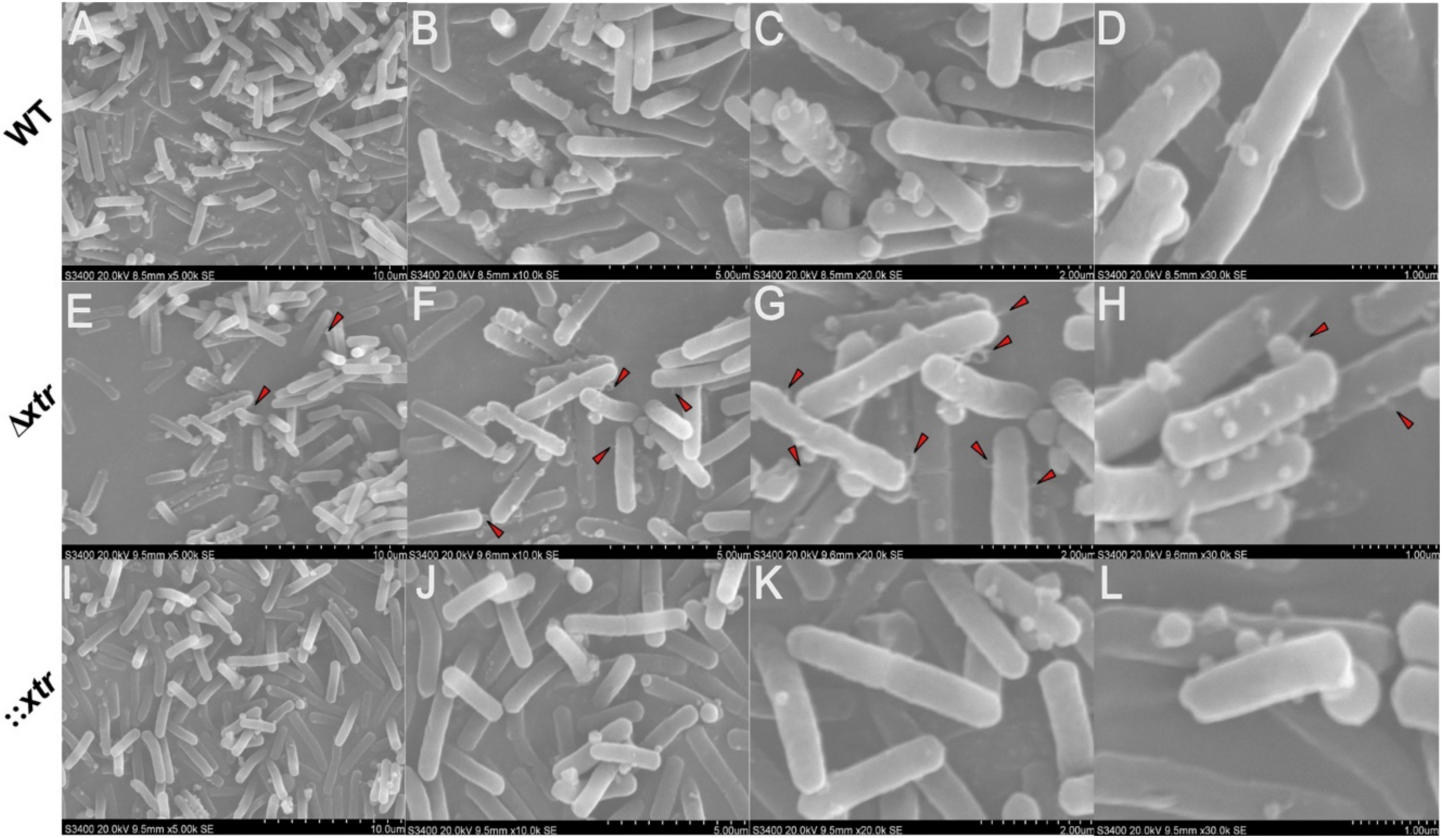
Surface morphology of WT, Δ*xtr* and Δ*xtr* strains. **(A–D)** Scanning electron microscopy (SEM) images of the wild-type (WT) strain at magnifications of 5,000× **(A)**, 10,000× **(B)**, 20,000× **(C)**, and 30,000× **(D)**. **(E–H)** SEM images of the Δ*xtr* strain at the same magnifications. **(I–L)** SEM images of the complement (::*xtr*) strain at the same magnifications. Arrows indicate filamentous attachments observed on the cell surface.

To investigate whether the reduced autolysis rate of the Δ*xtr* mutant is associated with changes in its cell surface structure (Figure 2), we examined the surface morphology of WT, Δ*xtr*, and ::*xtr* strains using scanning electron microscopy (SEM) (Figures 2A–L). SEM analysis revealed more filamentous attachments surrounding the Δ*xtr* knockout strain (Figures 2E–H) compared to the WT (Figures 2A–D) and ::*xtr* (Figures 2I–L) strains, which exhibited fewer filamentous attachments.

### Deletion of *xtr* downregulates TcdA/TcdB expression and cytotoxicity

**FIGURE 3:**
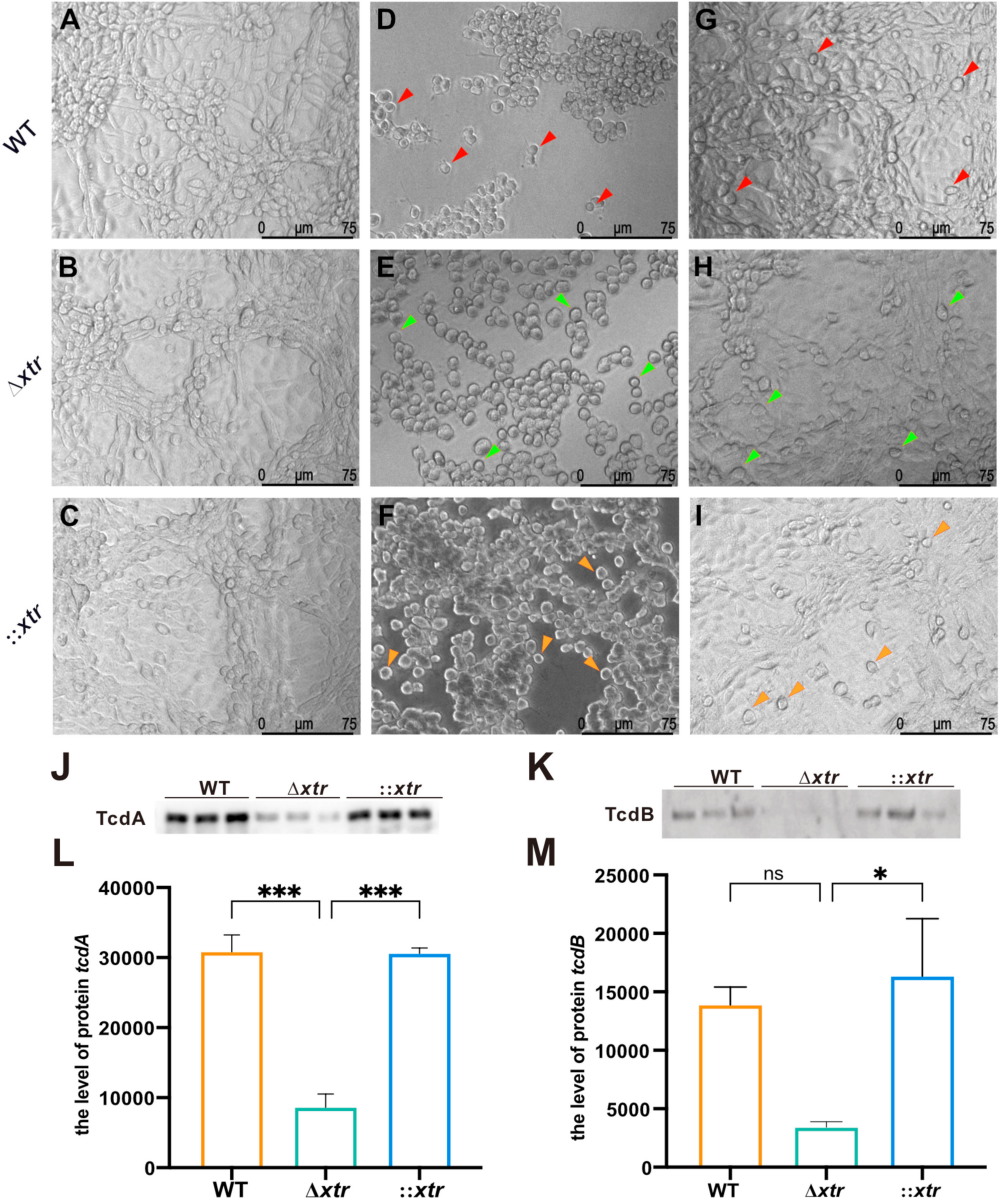
Cell toxicity of WT, Δ*xtr* and ::*xtr* strains. **(A–C)** Control Vero cells without toxin supplementation, exhibiting a spindle-shaped morphology. **(D–F)** Vero cells infected with undiluted culture supernatants from WT, Δ*xtr*, and ::*xtr* strains, showing rounding and detachment (indicated by red, green, and orange arrows). **(G–I)** Maximum dilution factor at which culture supernatants from WT **(G)**, Δ*xtr* **(H)**, and ::*xtr* **(I)** strains induced morphological changes in Vero cells. Red, green, and orange arrows indicate rounded cells. **(J–K)** Western blot analysis of TcdA **(J)** and TcdB **(K)** protein expression. **(L,M)** Quantitative analysis of TcdA **(L)** and TcdB **(M)** protein expression levels. Data are mean ± SEM (*n* = 3). “ns” indicates no significant difference, *P < 0.05, ***P < 0.001.

Preliminary phenotypic characterization of the Δ*xtr* mutant revealed downregulation of toxin gene expression (Figures 1B,C). To validate these findings and investigate its impact on cytotoxicity, the Vero cell model was employed to assess cytotoxicity and toxin protein levels (Figure 3). Untreated Vero cells (control group) exhibited typical morphology, characterized by regular spindle shapes, tight intercellular junctions, and well-defined boundaries (Figures 3A–C). Following treatment with undiluted culture supernatants from the wild-type (WT) strain, the Δ*xtr* mutant, and the ::*xtr* complemented strain, cells treated with WT and the complemented strain showed significant rounding, detachment, and disruption of tight junctions (Figures 3D–F). Quantitative analysis of cytotoxicity was performed based on the maximum dilution fold of the supernatant that induced cell rounding. The results showed that the WT strain and the ::*xtr* complemented strain maintained cytotoxic activity at a dilution of 0.5 × 10^8^ (Figures 3G,I), whereas the Δ*xtr* mutant exhibited significantly reduced cytotoxicity, showing activity only at a dilution of 0.5 × 10^5^ (Figure 3H). These findings indicate that the Δ*xtr* mutant has attenuated cytotoxicity, which can be restored by genetic complementation.

To elucidate the mechanism behind the reduced cytotoxicity observed in the Δ*xtr* strain, we analyzed protein expression levels of the key virulence factors TcdA and TcdB via Western blot. Results indicated a significant downregulation of both toxins in the Δ*xtr* strain (Figures 3J–M), with expression levels restored to WT levels in the ::*xtr* strain. These findings support the conclusion that the Xtr functions as a positive regulator of toxin expression in *C. difficile*.

Meanwhile, cell viability was assessed using the CCK-8 assay following exposure to culture supernatants of the WT, Δ*xtr* mutant, and ::*xtr* complemented strains (Supplementary Figure S5). Compared with the control group (Con), treatment with the WT *C. difficile* supernatant resulted in a significant decrease in cell viability. Notably, the Δ*xtr* mutant supernatant partially restored cell viability, which was significantly higher than that of the WT group (*P < 0.05). The ::*xtr* complemented strain exhibited cell viability comparable to that of the WT strain. Collectively, these results indicate that *xtr* is involved in the regulation of *C. difficile* virulence.

### Reduced hydrogen peroxide tolerance and antibiotic susceptibility in Δ*xtr* strain

**FIGURE 4:**
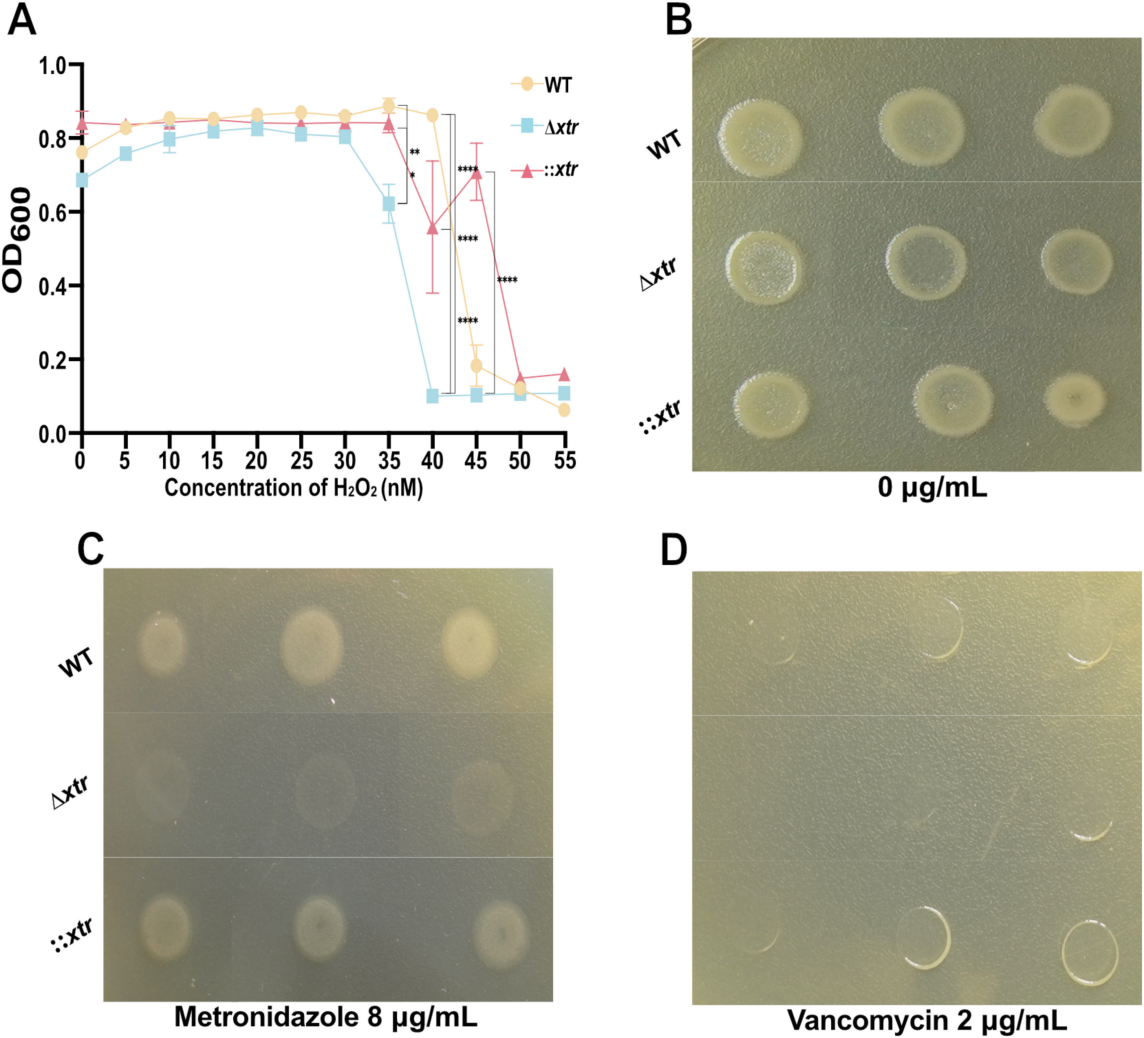
Tolerance of WT, Δ*xtr* and ::*xtr* to hydrogen peroxide and antibiotics. **(A)** Tolerance of WT, Δ*xtr*, and ::*xtr* strains to hydrogen peroxide. The x-axis represents H_2_O_2_ concentration (nM), and the y-axis represents OD_600_. Curves represent growth OD values for WT (yellow), Δ*xtr* (light blue), and ::*xtr* (red) strains. **(B–D)** Antibiotic susceptibility testing of WT, Δ*xtr*, and ::*xtr* strains: **(B)** 0 μg/mL (untreated control), **(C)** 8 μg/mL metronidazole, **(D)** 2 μg/mL vancomycin. Data are mean ± SEM (*n* = 3). *P < 0.05, **P < 0.01, ****P < 0.0001.

Given the roles of XRE family transcriptional regulators in oxidative stress resistance in other pathogens like *Streptomyces coelicolor*, *Streptococcus suis*, and *Enterococcus faecalis* (Hu et al., 2019; Long et al., 2022; Verneuil et al., 2005; Zhu et al., 2018), and the fact that first-line antibiotics for CDI—metronidazole and vancomycin—exert their effects, in part, by inducing ROS production, we hypothesized that the *xtr* gene might regulate oxidative stress response and antibiotic resistance in *C. difficile.* Metronidazole’s efficacy can be compromised by reduced ROS generation due to the loss or downregulation of specific reductase systems (nitroreductase-specific reductase system) (Boekhoud et al., 2020), while vancomycin’s bactericidal activity against *Staphylococcus aureus* is linked to ROS induction (Li et al., 2015). Therefore, we investigated the ROS tolerance and antibiotic susceptibility of WT, Δ*xtr*, and ::*xtr* strains (Figure 4).

Hydrogen peroxide tolerance assays showed that within the concentration range of 0–30 nM, the tolerance of WT, Δ*xtr*, and ::*xtr* strains exhibited only minor differences; however, when the concentration increased to 45–50 nM, the tolerance of the Δ*xtr* mutant was significantly lower than that of the WT and ::*xtr* strains (Figure 4A). This indicates that deletion of *xtr* significantly weakens oxidative stress tolerance, a phenotype restored, and even enhanced, by complementation in the ::*xtr* strain (Figure 4A).

Building on this finding, we examined the impact of *xtr* on susceptibility to metronidazole and vancomycin, both antibiotics whose mechanisms involve ROS-mediated bacterial killing (Card et al., 2025). The Minimum Inhibitory Concentration (MIC) of WT, Δ*xtr*, and ::*xtr* were determined for Metronidazole and Vancomycin. On the 0 μg/mL control plates, typical *C. difficile* colonies were formatted (Figure 4B). The Δ*xtr* strain exhibited a marked reduction in MIC compared to the WT strain for both agents. For metronidazole, the MIC of the Δ*xtr* (8 μg/mL) was half that of the WT strain (16 μg/mL) (Figure 4C). Similarly, for vancomycin, the MIC of the Δ*xtr* strain (2 μg/mL) was half that observed in the WT strain (4 μg/mL) (Figure 4D and Supplementary Table S4). According to the CLSI standard (Liebens et al., 2014), the Δ*xtr* strain shifted from intermediate to susceptible for metronidazole, confirming that *xtr* deletion weakens resistance to metronidazole. In contrast, the vancomycin MIC decreased in the Δ*xtr* strain, it remained within the resistant threshold range (Supplementary Table S4).

### Transcriptome analysis

**FIGURE 5:**
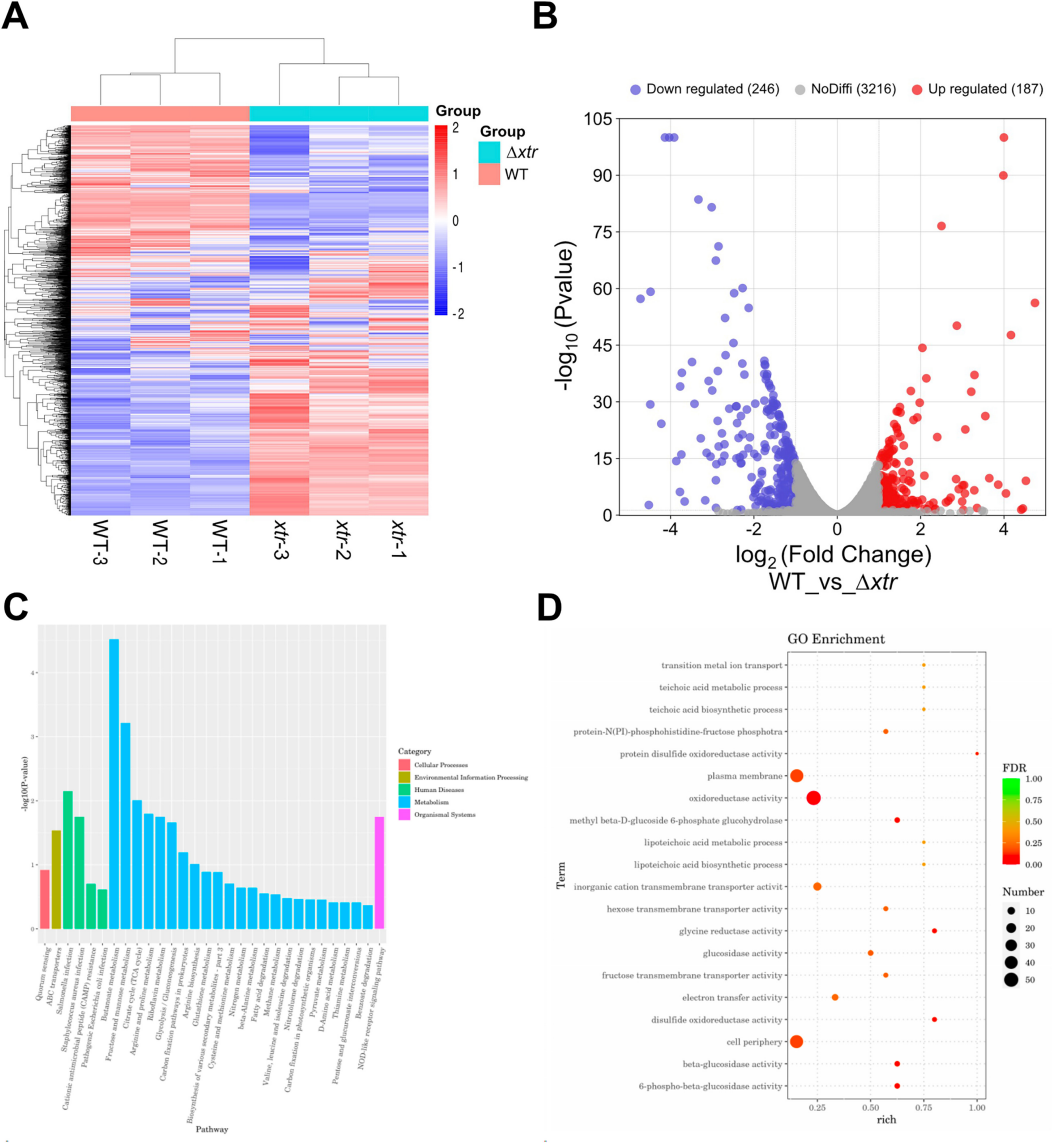
Transcriptomic analysis of WT and Δ*xtr* strains. **(A)** Hierarchical clustering heatmap of differentially expressed genes. Columns represent individual samples (strains), and rows represent gene clusters. Pink indicates upregulated genes, and blue indicates downregulated genes. Genes with similar expression patterns are clustered together. **(B)** Volcano plot of differentially expressed genes. The x-axis shows the log_2_ fold change in expression, and the y-axis shows the negative log_10_ of the p-value. Purple dots represent downregulated genes, red dots represent upregulated genes, and gray dots represent genes with no significant differential expression. **(C)** KEGG pathway enrichment analysis of differentially expressed genes. The x-axis represents enriched KEGG pathways, and the y-axis shows the negative log_10_ of the *p*-value. Red bars represent cellular processes, dark green bars represent environmental information processing, green bars represent human diseases, blue bars represent metabolism, and pink bars represent organismal systems. Bar height indicates the number of differentially expressed genes annotated to the pathway. **(D)** GO functional enrichment bubble plot. The y-axis represents enriched biological process GO terms, and the x-axis indicates the enrichment ratio. Bubble size represents the number of differentially expressed genes annotated to the term, and color intensity corresponds to the false discovery rate (FDR; green = low FDR, red = high FDR).

We employed RNA sequencing to compare gene expression profiles between the WT and Δ*xtr* strains (Figure 5). Differential gene expression analysis was performed using DESeq, with criteria of *P* < 0.05 and | log_2_FoldChange| > 1 to define significantly differentially expressed genes. Statistical analysis of sample data and generation of a heatmap (Figure 5A) and volcano plot (Figure 5B) were performed using the R package ggplot2 (version 2.4-6). Analysis revealed 246 downregulated genes and 187 upregulated genes in the Δ*xtr* mutant compared to the WT, with 3216 genes showing no significant change in expression (the top 10 genes with the highest fold change in upregulation and downregulation are presented in Supplementary Tables S5, S6). Consistent with our RT-qPCR results, *xtr* expression was barely detectable.

KEGG pathway enrichment analysis indicated that differentially expressed genes were annotated to 68 known KEGG pathways (Figure 5C). Metabolic pathways comprised the largest functional category, encompassing 23 unigenes involved in pathways such as butyrate metabolism, fructose and mannose metabolism, the tricarboxylic acid (TCA) cycle, arginine and proline metabolism, riboflavin metabolism, glycolysis/gluconeogenesis, prokaryotic carbon fixation, arginine biosynthesis, glutathione metabolism, biosynthesis of secondary metabolites (part III), cysteine and methionine metabolism, nitrogen metabolism, β-alanine metabolism, fatty acid degradation, methane metabolism, valine, leucine and isoleucine degradation, nitrotoluene degradation, photosynthetic carbon fixation, pyruvate metabolism, D-amino acid metabolism, thiamine metabolism, pentose and glucuronate interconversions, and benzoate degradation (Figure 5C, blue). The second most represented category was “Human Diseases,” containing 4 unigenes related to *Salmonella* infection, *Staphylococcus aureus* infection, cationic antimicrobial peptide (CAMP) resistance, and pathogenic *E. coli* infection (Figure 5C, green). Furthermore, quorum sensing, ABC transporters, and NOD-like receptor signaling pathways were enriched within the “Cellular Processes” (Figure 5C, red), “Environmental Information Processing” (Figure 5C, yellow), and “Organismal Systems” (Figure 5C, magenta) categories, respectively. Notably, the TCA cycle, pyruvate metabolism, amino acid degradation, and pathways related to nitrogen/sulfur and glutathione metabolism, collectively point towards a role for iron-sulfur proteins (ferredoxins, Fd). Further Gene Ontology (GO) analysis (Figure 5D) revealed a highly significant enrichment of oxidoreductase activity. *4Fe-4S_BP*, which encodes a 4Fe-4S-binding domain protein, exhibits a highly conserved β-barrel fold typical of this protein class and contains a well-defined, structurally stable pocket for coordinating iron-sulfur clusters (Supplementary Figure S6). This gene was downregulated 3.69-fold in the Δ*xtr* mutant compared to the WT, potentially contributing to the observed decrease in ROS tolerance.

### Xtr modulates ROS and antibiotic resistance through regulation of *4Fe-4S_BP*

**FIGURE 6:**
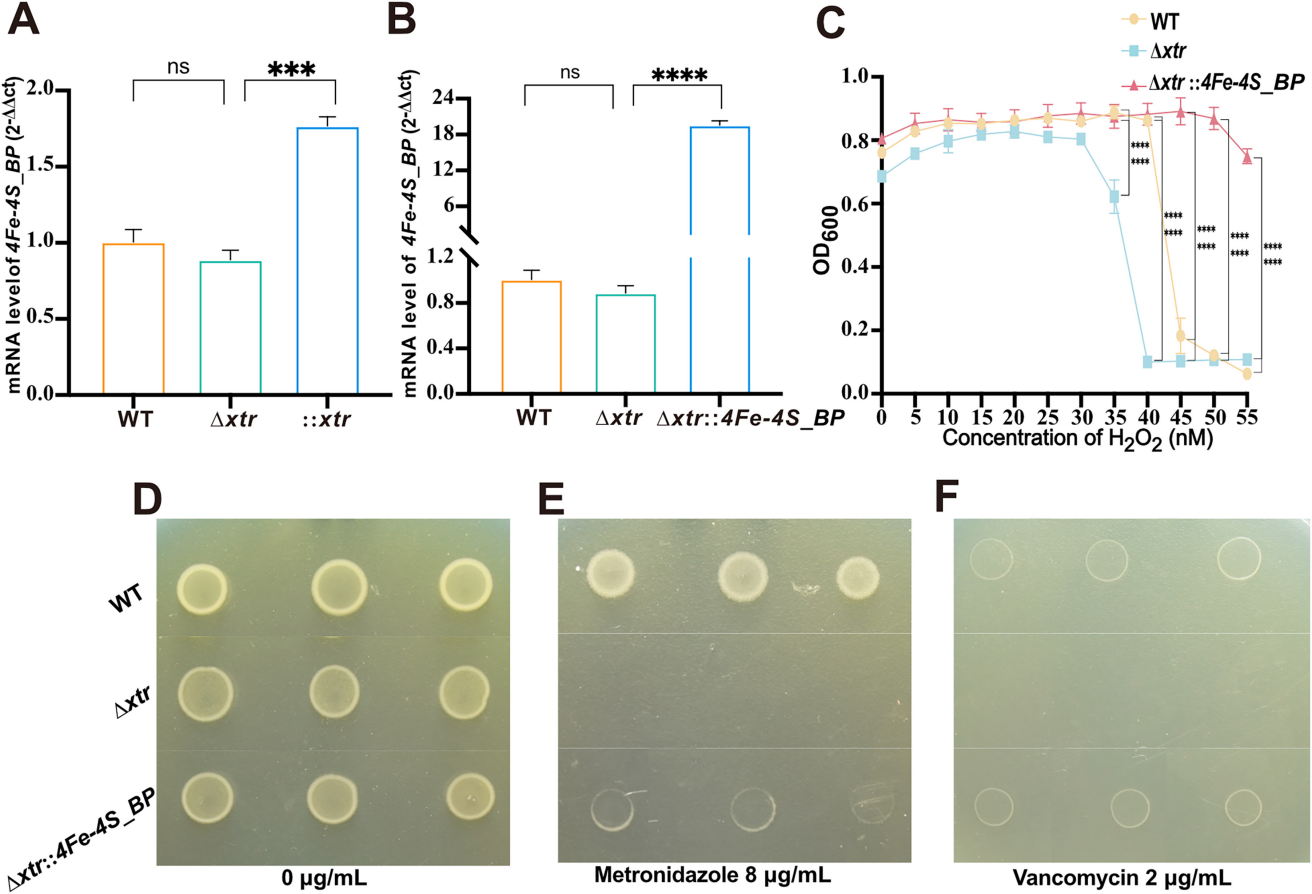
Restoration of *C. difficile* tolerance to ROS and antibiotics by overexpression of *4Fe-4S_BP*. **(A)** RT-qPCR analysis of *4Fe-4S_BP* expression levels in WT, Δ*xtr*, and ::*xtr* strains. **(B)** *4Fe-4S_BP* expression levels in WT, Δ*xtr*, and Δ*xtr* complemented with the *4Fe-4S_BP* (Δ*xtr*::*4Fe-4S_BP*). **(C)** Growth curves of different strains under varying concentrations of hydrogen peroxide (H_2_O_2_). The x-axis represents H_2_O_2_ concentration (nM), and the y-axis represents optical density at 600 nm (OD_600_). **(D–F)** Antibiotic susceptibility testing of WT, Δ*xtr*, and Δ*xtr*::*4Fe-4S_BP* strains: **(D)** 0 μg/mL (untreated control), **(E)** 8 μg/mL metronidazole, **(F)** 2 μg/mL vancomycin. Data are mean ± SEM (*n* = 3). “ns” indicates no statistically significant difference. ***P ≤ 0.001, ****P ≤ 0.0001.

4Fe-4S cluster-containing proteins are a widely distributed and functionally diverse class of proteins characterized by their ability to bind to a cubic iron-sulfur cluster consisting of four iron atoms and four inorganic sulfur atoms. These proteins mediate crucial biological functions including electron transfer, enzymatic catalysis, gene regulation, and environmental signal perception. Recent research has illuminated their structural diversity, the complexity of their assembly mechanisms, and their central role in cellular metabolism and homeostasis (Cheng et al., 2020; Gray et al., 2023; Wachnowsky et al., 2019). To validate the impact of the regulatory relationship between Xtr and 4Fe-4S-binding domain proteins on *C. difficile* ROS tolerance, we overexpressed the *4Fe-4S_BP* in the Δ*xtr* knockout strain (Δ*xtr*::*4Fe-4S_BP*) and assessed the expression levels of the *4Fe-4S_BP* in WT, Δ*xtr*, ::*xtr*, and Δ*xtr*::*4Fe-4S_BP* strains using RT-qPCR, followed by additional antibiotic tolerance assays (Figure 6). Consistent with our transcriptomic data, expression of the *4Fe-4S_BP* was downregulated in the Δ*xtr* strain, while expression was restored to WT levels in the ::*xtr* complementation strain (Figure 6A). Overexpression of the *4Fe-4S_BP* in the Δ*xtr* strain resulted in significantly higher expression levels compared to the WT strain (Figure 6B). We then evaluated the ROS tolerance of the Δ*xtr*::*4Fe-4S_BP* strain, demonstrating that the WT strain survived exposure to hydrogen peroxide concentrations ranging from 0 to 45 nM, whereas the Δ*xtr* knockout strain exhibited reduced tolerance, surviving only up to 40 nM. Importantly, the Δ*xtr*::*4Fe-4S_BP* overexpression strain remained viable at a concentration of 55 nM hydrogen peroxide (Figure 6C).

Metronidazole and vancomycin are known to induce the production of endogenous ROS in *C. difficile*, leading to oxidative damage (e.g., to DNA, proteins, and iron-sulfur clusters), and triggering a global stress response for survival (Turner et al., 2022). We hypothesized that reconstitution of 4Fe-4S binding protein levels would enhance ROS tolerance in *C. difficile*, thereby increasing resistance to antibiotics that function via oxidative stress. Consequently, we investigated whether the Δ*xtr*::*4Fe-4S_BP* strain exhibited restoration of resistance to metronidazole and vancomycin. On the 0 μg/mL control plates, all strains grew normally (Figure 6D), As shown in Figures 6E,F, the Δ*xtr* strain displayed significantly reduced resistance to both metronidazole and vancomycin—with MIC values decreasing to 8 and 2 μg/mL, respectively—compared to the WT strain. Overexpression of the *4Fe-4S_BP* in the Δ*xtr*::*4Fe-4S_BP* strain restored resistance levels to those observed in the WT strain (Supplementary Table S7). These results suggest that Xtr likely targets the expression of *4Fe-4S_BP*, thereby influencing *C. difficile*’s tolerance to both ROS and antibiotics.

### Screen of Xtr promoter binding sites

**FIGURE 7:**
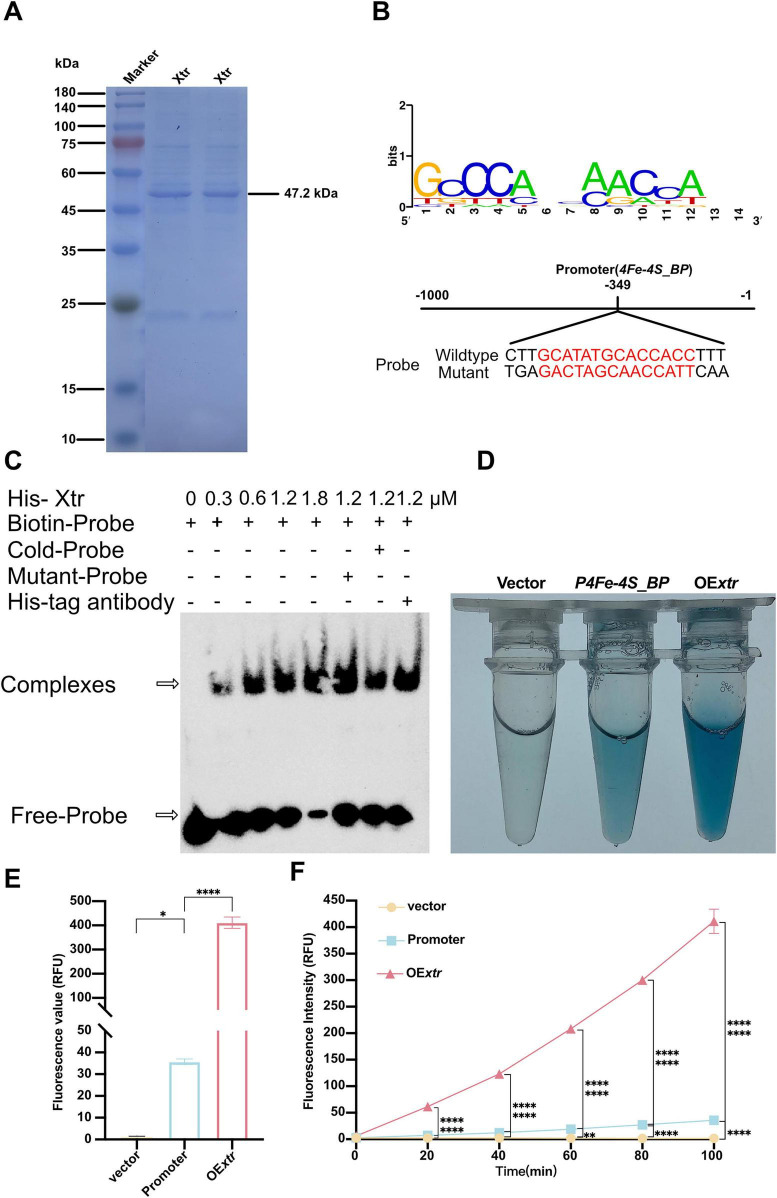
Expression, DNA-binding activity of Xtr protein, and its regulatory effect on the *4Fe-4S_BP* promoter. **(A)** SDS-PAGE analysis of purified recombinant His-tagged Xtr protein. The purified Xtr migrates at the expected molecular weight of ∼47.2 kDa, consistent with its theoretical size. Marker, protein molecular weight marker; Xtr, purified His-Xtr protein. **(B)** The consensus binding motif of Xtr identified by MEME analysis, and the design of wild-type and mutant biotin-labeled probes targeting the binding site in the *4Fe-4S_BP* promoter region (position -349). **(C)** EMSA demonstrating the direct and specific binding of Xtr to the *4Fe-4S_BP* promoter. Purified His-Xtr was incubated with biotin-labeled wild-type probe at increasing concentrations (0–2.1 μM). In the competition experiment, excess amounts of unlabeled wild-type probe (Cold-Probe) or mutant probe (Mutant-Probe) were added, respectively. An anti-His-tag antibody was also tested; no supershift was observed under the conditions used. The arrow indicates the Xtr-probe complexes, with the position of the free probe as indicated in the figure. **(D)** *gusA* reporter gene staining results for different treatment groups. From left to right: empty vector control (vector), *4Fe-4S_BP* promoter group (P*4Fe-4S_BP*), and Xtr overexpression group (OE*xtr*). Blue intensity reflects phenotypic differences in the strains. **(E)** Quantitative analysis of fluorescence values for different treatment groups. The y-axis represents relative fluorescence units (RFU). **(F)** Time course of fluorescence intensity for different treatment groups. The x-axis represents incubation time (minutes), and the y-axis represents fluorescence intensity (RFU). Data are mean ± SEM (*n* = 3). *P ≤ 0.05, ****P ≤ 0.0001.

To identify the DNA sequence to which Xtr binds, we hypothesized that Xtr directly regulates the expression of the *4Fe-4S_BP* by binding to its promoter regions (Figure 7). Motif analysis using the MEME suite revealed a 14-bp potential Xtr binding motif (5’-GCATATGCACCACC-3’) within the promoter regions of *4Fe-4S_BP*. To validate this prediction, we first expressed and purified recombinant Xtr protein *in vitro* (Figure 7A). Subsequently, MEME motif analysis was used to identify a potential binding site in the promoter region (at position -349) of the *C. difficile 4Fe-4S_BP*. Based on this site, wild-type and mutant biotin-labeled probes were designed, with the mutant probe containing base substitutions in the core binding motif for subsequent specificity validation (Figure 7B). EMSA revealed a concentration-dependent binding of Xtr to the *4Fe-4S_BP* promoter probe. Unlabeled cold probe competition effectively decreases this binding, whereas the mutant probe failed to compete for binding. Collectively, these results confirm the specific interaction between Xtr and the *4Fe-4S_BP* promoter (Figure 7C and Supplementary Figure S7).

Next, we investigated the functional impact of Xtr binding on *4Fe-4S_BP* promoter activity using a *gusA* reporter assay. The *4Fe-4S_BP* promoter was cloned into the pMTL82151-*gusA* plasmid, and a co-expression vector for *xtr* was constructed. Following transformation into *E. coli* BL21 cells, X-Gluc chemical staining showed weak blue coloration with the *4Fe-4S_BP* promoter alone, but significantly increased coloration when co-expressed with *xtr* (Figure 7D). This observation was corroborated by fluorometric measurement of GUS enzyme activity, which demonstrated a significantly higher signal in the *xtr* co-expression group compared to the group containing only the *4Fe-4S_BP* promoter (Figures 7E,F). These results collectively confirm that 5’-GCATATGCACCACC-3’ is a specific binding site for the Xtr transcription factor and that Xtr protein directly activates transcription of the *4Fe-4S_BP* promoter through binding to this site.

### Mechanism of Xtr Regulation of 4Fe-4S binding Protein

**FIGURE 8:**
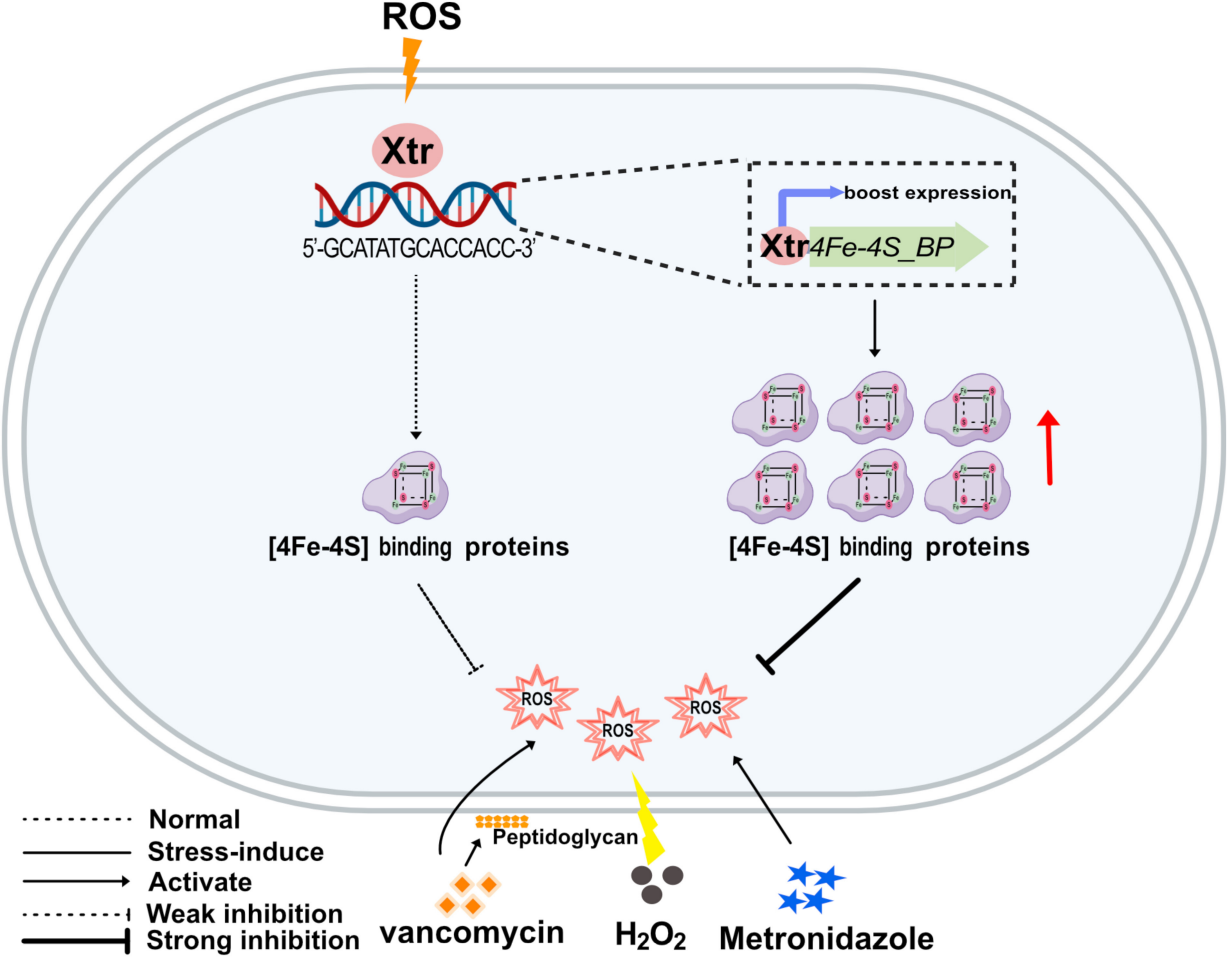
Mechanism of Xtr Regulation of 4Fe-4S binding Protein. Under normal physiological conditions (dashed lines), Xtr constitutively activates the expression of a basal level of 4Fe-4S cluster-binding protein, which exert weak inhibitory effects on intracellular ROS levels induced by vancomycin, H_2_O_2_, and metronidazole. Upon ROS stress (solid lines), Xtr is further activated to boost its own expression, leading to a significant upregulation of [4Fe-4S] cluster-binding protein. This enhanced expression results in a strong inhibitory effect on intracellular ROS accumulation, thereby conferring increased tolerance to oxidative stress and antibiotic exposure in *C. difficile*.

Within *C. difficile* cells, the transcriptional regulator Xtr specifically binds to a 5’-GCATATGCACCACC-3’ cis-regulatory element within the promoter regions of its target genes, thereby promoting the expression of 4Fe-4S binding protein. Under non-stressed conditions (e.g., in the absence of oxidative stress from hydrogen peroxide or antibiotics), Xtr levels are maintained at a low baseline, resulting in low expression of 4Fe-4S binding protein. However, upon exposure to oxidative and antibiotic stresses, *xtr* mRNA levels were upregulated in *C. difficile* (Supplementary Figure S8). Xtr then binds to the upstream promoter sequences of *4Fe-4S_BP*, activating its transcription and enhancing its expression. The 4Fe-4S binding protein enhances intracellular redox capacity, promotes ROS scavenging, and maintains the stability of proteins harboring 4Fe-4S clusters, ultimately increasing *C. difficile*’s tolerance to oxidative stress (Figure 8).

## Discussion

This study identifies Xtr as a previously uncharacterized XRE family regulator that contributes to stress adaptation in *C. difficile*. Deletion of *xtr* reduced tolerance to hydrogen peroxide, lowered resistance to metronidazole and vancomycin, decreased *tcdA* and *tcdB* expression and cytotoxicity, and reduced autolysis. Transcriptomic analysis further showed marked downregulation of the 4Fe-4S binding protein gene *4Fe-4S_BP* in the *xtr* deletion mutant. Together with the EMSA, reporter assay, and rescue experiment in which *4Fe-4S_BP* overexpression restored key phenotypes in the *xtr* deletion background, these findings support a model in which *4Fe-4S_BP* is a major downstream effector of Xtr. More broadly, the data suggest that Xtr links Fe-S homeostasis with oxidative adaptation, antibiotic response, and virulence-associated outputs in *C. difficile*.

A central point in interpreting these data is the distinction between proteins that harbor a 4Fe-4S cluster and proteins that bind or traffic 4Fe-4S clusters. Proteins in the first category use the cluster as an intrinsic catalytic or structural cofactor, whereas proteins in the second category typically participate in cluster assembly, transfer, stabilization, or homeostasis. This distinction is well established in the Fe-S field and is important here because it argues against equating *4Fe-4S_BP* with a conventional Fe-S enzyme whose own activity simply depends on a bound cluster (Cheng et al., 2020; Imlay, 2006; Saninjuk et al., 2019). Accordingly, our data do not demonstrate that *4Fe-4S_BP* directly repairs oxidized Fe-S clusters. A more cautious interpretation is that Xtr dependent upregulation of *4Fe-4S_BP* helps preserve Fe-S homeostasis under oxidative challenge, thereby limiting the downstream consequences of cluster damage. This is also consistent with work showing that oxidized Fe-S clusters can, in some contexts, be restored by cellular reduction and remetallation systems, but that protection often depends on preventing irreversible damage and maintaining cluster homeostasis rather than on a single dedicated repair reaction (Djaman et al., 2004; Lu and Imlay, 2019).

The transcriptomic data support this model at the systems level. In addition to the 3.69-fold decrease in *4Fe-4S_BP*, the *xtr* deletion mutant showed broader changes in genes related to oxidoreductase activity and metabolic pathways involving central carbon, amino acid, and nitrogen or sulfur metabolism. These findings are biologically relevant because Fe-S proteins are deeply integrated into redox balance, metabolic flux, and stress adaptation, and bacteria commonly coordinate Fe-S homeostasis with broader transcriptional responses through regulators such as IscR and SufR (Cheng et al., 2020; Saninjuk et al., 2019). Thus, loss of Xtr likely does not affect only a single protective factor; rather, it appears to alter the metabolic context in which oxidative stress is encountered. At the same time, the rescue of major stress-related phenotypes by *4Fe-4S_BP* overexpression indicates that this gene is not simply a passive marker of transcriptional disturbance, but a functionally important branch of the Xtr regulon.

The oxidative stress phenotype is also mechanistically plausible in light of current knowledge about Fe-S chemistry. Fe-S clusters are among the best known intracellular targets of oxygen and reactive oxygen species, and their disruption can compromise enzyme activity while also contributing to the generation of redox active iron that exacerbates oxidative injury (Imlay, 2006; Kint et al., 2022). In this framework, Xtr dependent activation of a 4Fe-4S binding protein may strengthen peroxide tolerance not because *4Fe-4S_BP* is proven to directly repair damaged clusters, but because it may facilitate cluster preservation, cluster reassembly, or safe handling of iron during stress. Our data therefore support a protective homeostasis model, but the exact biochemical function of *4Fe-4S_BP* remains to be established experimentally.

The antibiotic phenotypes are also interpretable within this framework, although they require some caution. The metronidazole result is particularly coherent because metronidazole activity in anaerobes depends on reductive activation and cellular redox metabolism, and metronidazole resistance in *C. difficile* has repeatedly been linked to metabolic state and electron transfer pathways (Boekhoud et al., 2020; Xu et al., 2022). Perturbation of Fe-S homeostasis could therefore affect metronidazole susceptibility by altering intracellular redox balance or the ability to withstand radical mediated injury. The vancomycin phenotype is less straightforward. Although ROS associated effects of vancomycin have been reported in some organisms and conditions, especially in *Staphylococcus aureus*, the idea that bactericidal antibiotics share a universal ROS based killing mechanism remains controversial (Keren et al., 2013; Li et al., 2015; Liu and Imlay, 2013). For that reason, our results should not be interpreted as evidence that vancomycin acts through the same oxidative mechanism as metronidazole in *C. difficile*. A more conservative explanation is that Xtr affects vancomycin susceptibility indirectly, possibly through changes in cell envelope physiology, stress buffering, or peptidoglycan turnover.

The reduced autolysis of the *xtr* deletion mutant is consistent with that interpretation. Because deletion of *xtr* did not substantially impair overall growth, the decreased Triton X-100-induced autolysis is unlikely to reflect a general proliferation defect. Instead, it more likely indicates altered cell envelope architecture or reduced activity of autolysins involved in cell wall remodeling. This is reasonable in *C. difficile*, where peptidoglycan hydrolases are known to contribute to autolysis, cell wall turnover, and toxin release (Dhalluin et al., 2005; El Meouche and Peltier, 2018; Wydau-Dematteis et al., 2018). Therefore, the lower autolysis observed here may help explain both the toxin-associated phenotype and part of the vancomycin phenotype. By contrast, although a more filamentous attachments was observed around the *xtr* deletion mutant, this finding was not quantitatively validated and therefore should not be overinterpreted as a distinct morphological defect. Instead, when considered alongside the reduced autolysis phenotype, these observations may suggest subtle changes in cell-surface properties or envelope homeostasis. Such changes could be related to altered cell wall turnover or autolysin-associated processes, but this will require further experimental validation.

The virulence-related findings further suggest that Xtr coordinates stress adaptation with pathogenic potential. The *xtr* deletion mutant showed reduced *tcdA* and *tcdB* expression, lower toxin protein levels, and diminished cytotoxicity, indicating that Xtr positively influences toxin-associated outputs. The transcriptome did not fully mirror the magnitude of all toxin-related changes seen in targeted assays, but this discrepancy is not unexpected. Toxin production in *C. difficile* is highly dynamic and is tightly integrated with nutrient sensing, redox state, and broader metabolic regulation through factors such as TcdR, CodY, CcpA, Rex, and other global regulators (Bouillaut et al., 2015; Bouillaut et al., 2019; Majumdar and Govind, 2022). Thus, the toxin phenotype likely reflects both direct and indirect consequences of Xtr-dependent regulation, with altered Fe-S homeostasis and metabolic remodeling feeding into established virulence networks.

Comparison with previous work also highlights the functional diversity of XRE family regulators and suggests that Xtr represents a distinct regulatory logic rather than a simple extension of previously described systems. In *Streptococcus suis*, SrtR and XtrSs have been linked to oxidant tolerance and virulence, but the validated downstream outputs differ from those identified here. One XtrSs study connected peroxide fitness to repression of a downstream surface associated gene, whereas a later study showed that XtrSs represses *argR* and thereby modulates the arginine deiminase system, acid resistance, and survival against macrophage-associated stress (Chen et al., 2024; Hu et al., 2019; Zhang et al., 2022). Outside *streptococci*, BioX represses biotin biosynthesis in *Riemerella anatipestifer*, NceR activates *ompA* in *Neisseria gonorrhoeae* in an iron responsive context, LfsT coordinates phage lytic development and host metabolism in *Pseudomonas aeruginosa*, and DdiA participates in a noncanonical DNA damage response in *Myxococcus xanthus* (Holley et al., 2023; Jung et al., 2025; Long et al., 2022; Ren et al., 2021). These studies collectively show that XRE regulators can function either as activators or repressors and can control very different physiological modules. The present study extends that diversity by identifying an XRE regulator in *C. difficile* that directly activates *4Fe-4S_BP* and thereby links Fe-S homeostasis to stress adaptation.

Several limitations should be acknowledged. First, while the peroxide-associated phenotype exhibited a statistically significant reduction, the magnitude of the actual fold change was relatively small, suggesting that Xtr is likely one component of a broader oxidative defense network rather than a dominant peroxide regulator (Kint et al., 2022). Second, only one direct target, *4Fe-4S_BP*, was experimentally validated, so the full Xtr regulon remains unresolved. Third, the current data do not establish whether Xtr itself directly senses oxidative cues or whether its activity is modulated by another upstream signal. Fourth, the mechanism by which *4Fe-4S_BP* protects the cell remains inferential; direct biochemical evidence for cluster transfer, iron buffering, or promotion of Fe-S repair is still lacking. Finally, all experiments were conducted *in vitro*, so the contribution of the Xtr-*4Fe-4S_BP* axis during infection remains to be determined.

Future work should therefore define the broader Xtr regulon by integrating transcriptomics with genome-wide DNA-binding analysis, determine whether Xtr undergoes redox-dependent structural or DNA-binding changes, and biochemically characterize *4Fe-4S_BP* to distinguish among cluster trafficking, cluster stabilization, and iron-buffering functions. It will also be important to test how Xtr influences autolysin activity, cell envelope composition, and toxin release, and whether these effects contribute to antibiotic susceptibility independently of oxidative stress tolerance. Finally, infection models and physiologically relevant stress conditions will be needed to establish the role of this pathway in intestinal survival and disease. Overall, the present study supports a model in which Xtr directly activates *4Fe-4S_BP*, thereby promoting Fe-S homeostasis-associated stress fitness in *C. difficile*, revealing a new regulatory layer that connects redox adaptation with antibiotic response and virulence-related phenotypes.

## Conclusion

In conclusion, this study identifies Xtr as a previously uncharacterized XRE family transcriptional regulator that contributes to oxidative stress adaptation, antibiotic response, and virulence associated phenotypes in *C. difficile*. We demonstrate that Xtr directly binds to the promoter of the *4Fe-4S_BP* and activates its transcription. Loss of *xtr* reduced hydrogen peroxide tolerance, altered susceptibility to metronidazole and vancomycin, decreased autolysis, and lowered toxin expression and cytotoxicity, whereas restoration of *4Fe-4S_BP* expression in the *xtr* deletion background rescued key stress related phenotypes. These findings support the existence of an Xtr-*4Fe-4S_BP* regulatory axis that links Fe-S homeostasis with bacterial fitness under stress. More broadly, this work expands current understanding of how XRE family regulators function in *C. difficile* and suggests that Xtr acts as part of a regulatory network coordinating redox balance, envelope associated physiology, and pathogenic potential. Although the precise biochemical role of *4Fe-4S_BP* and the full extent of the Xtr regulon remain to be defined, the present study provides direct experimental evidence for a novel transcriptional mechanism underlying stress adaptation in this pathogen. These results offer a useful framework for future studies of Fe-S dependent stress defense and may help identify new targets for controlling *C. difficile* survival and virulence.

## Data Availability

The datasets presented in this study can be found in online repositories. The names of the repository/repositories and accession number(s) can be found at: https://www.ebi.ac.uk/arrayexpress/, E-MTAB-15575.

## Footnotes

1
https://www.ncbi.nlm.nih.gov/

2
https://blast.ncbi.nlm.nih.gov/Blast.cgi

3
https://www.genome.jp/tools-bin/clustalw

4
https://services.healthtech.dtu.dk/services/TMHMM-2.0/

5
http://www.softberry.com/berry.phtml?topic=fprom&group=help&subgroup=promoter

6
https://meme-suite.org/meme/tools/meme
