# Supplementary material for: Xtr-mediated regulation of 4Fe-4S binding protein gene confers resistance to oxidative and antibiotic stress in Clostridioides difficile
Source: Front Microbiol. 2026 May 29;17:1817884. doi: 10.3389/fmicb.2026.1817884 (PMC13260099; doi:10.3389/fmicb.2026.1817884)
Supplement: Supplementary file 1 [file Supplementary_file_1.docx]

**Table S1** Strains and plasmids.

| Strains or Plasmids | Relevant characteristics | Reference or source |  |  |
| --- | --- | --- | --- | --- |
| Strains | | |  |  |
| NEB Express Competent *E.coli* (High efficiency) | General cloning host for plasmid manipulation | NEB |  |  |
| *E. coli* CA434 | Donor strain for conjugation between *E. coli* and *Streptomyces* | Lab stock |  |  |
| *Clostridium difficile* |  |  |  |  |
| *C.difficile 630* | Wild-type *Clostridioides difficile* 630 strain (CD630) | American Culture Collection, ATCC |  |  |
| Δ*pyrF* | *pyrF* gene disruption mutant, derived from CD630 | This work |  |  |
| Δ*pyrF*Δ*xtr* | *pyrF* gene and *xtr* gene disruption mutant, derived from Δ*pyrF* | This work |  |  |
| Δ*xtr* | *pyrF* gene complemented strain, derived from Δ*pyrF*Δ*xtr* | This work |  |  |
| ::*xtr* | *pyrF* and *xtr* complemented strain, derived from Δ*pyrF*Δ*xtr* | This work |  |  |
|  |  |  |  |  |
| Plasmids |  |  |  |  |
| pMTL82151 | pBP1 ori, CmR, ColE1 ori, TraJ, lacZ α fragment | [a] |  |  |
| pJJB1 | For deletion of the *pyrF* gene, containing upstream and downstream homologous arms of *pyrF*, derived from pMTL82151 | This work |  |  |
| pJJB-DE | Derived from pMTL82151, containing upstream and downstream homologous arms of *pyrF* from *Clostridium beijerinckii* NCIMB 8052 | This work |  |  |
| pJJB-DE1 | Derived from pMTL-DE, for deletion of the *xtr* gene, containing upstream and downstream homologous arms of *xtr* | This work |  |  |
| pJJBC1 | Derived from pMTL82151, for complement for *pyrF* gene, containing *pyrF* gene and promoter. | This work |  |  |
| pJJBC2 | Derived from pMTL-BJ2, for complement for *xtr* gene, containing *xtr* gene and promoter. | This work |  |  |
| pWWJ1 | Overxpression of the *4Fe-4S* gene in the ∆*xtr* knockout strain | This work |  |  |
| pWWJ2 | The *xtr* gene was inserted into the *pET-28a* vector for protein expression | This work |  |  |
| pWWJ3 | The promoter of the *4Fe-4S* gene was ligated into the pMTL82151 vector using *gusA* as the reporter gene to construct the 4Fe-4S gene promoter reporter plasmid | This work |  |  |
| pWWJ4 | Using *gusA* as the reporter gene, the promoter of the *xtr* gene was ligated into the pMTL82151 vector to construct the *xtr*-*4Fe-4S* gene promoter reporter plasmid | This work |  |  |

**Table S2** Primers used in this study.

| **Primers** | **Primer sequence (5′–3′)** |
| --- | --- |
| HW731 | GCCTGCAGACATGCAAGCATGCAAACAGTGCAAAAAAT |
| HW732 | GTTATAGGTCTTCCAACAACAGCTTTATCAAATTCGTCAGT |
| HW733 | ACTGACGAATTTGATAAAGCTGTTGTTGGAAGACCTATAACAA |
| HW734 | ACGACGGCCAGTGCCAAGCCCTGGGCCATTAGATAATAAG |
| HW735 | ACGACGGCCAGTGCCAAGCTATTGGTCCTGACCCTAATAC |
| HW736 | AGACTGGGAAAGATATCTCAT |
| HW681 | ATCAGGAAACAGCTATGACCG |
| HW682 | GTTTTCCCAGTCACGACGTT |
| HW601 | TTTTTTGTTACCCTAAGTTTTAAAACTAGACACTAATGATTGC |
| HW602 | AGATTATCAAAAAGGAGTTTTTATATGTTCTTCACTGCTTCT |
| HW605 | ATCGTAGAAATACGGTGTTT |
| HW606 | ACGTTAAGGGATTTTGGTCA |
| HW621 | CGAGGCCTGCAGACATGCAGGAAACAGGCAAAAAAATAG |
| HW622 | ATTTTTCCGTCAGTATGGGACACTAGAACCCCTTTATCCA |
| HW623 | TGGATAAAGGGGTTCTAGTGTCCCATACTGACGGAAAAAT |
| HW624 | CGACGGCCAGTGCCAAGCTTGAATGGCGTATTCCTCGTA |
| HW635 | TTAACCCCCCGTATCTAACA |
| HW636 | ATAGAAGTGTATTCGTCAACC |
| HW639 | CGAAATACACTCTTCCTGTC |
| HW721 | CGAGGCCTGCAGACATGCAAAAACTGATGGTTTCTTCAAG |
| HW722 | CGACGGCCAGTGCCAAGCTAAAAATGTTCTCCTTTCTGT |
| HW554 | GGGAGACTTGAGTGCAGGAG |
| HW555 | GTGCCTCAGCGTCAGTTACAGT |
| HW691 | CGTGGGTGCAGACAATTTGAAAGC |
| HW692 | ACTAGCCACCAAAGCGTCCATAAAG |
| HW949 | AGGAGAAGGAGCTACACGAAGACG |
| HW950 | GGGTCACCAAGAGGAGCTACTTTTC |
| HW1049 | GACCAGCATTTGCCAGTGTTTTAC |
| HW1050 | GACCAGCATTTGCCAGTGTTTTAC |
| HW2218 | GAATTCGAGCTCCGACAATTGGAGGAATTTCACTGT |
| HW2219 | CTCGAGTGCGGCCGCAAGCTTTATTTTTTGTTCGTTATC |
| HW2494 | GTGAAATTCCTCCAATAATCTATTTATCTCCTCTCTATCTCCTATCTCRA |
| HW2495 | GATTATTGGAGGAATTTCACTGAATGAAC |
| HW2496 | CGTCGACTCTAGAGGATCCTR]TAGTGGTGATGGRGATGATGTTTTTTGTTCGTTA |
| HW2540 | GTATGACCTTGCATATCACCACCTTTTTCAACAGCT |
| HW2541 | AGCTGTTGAAAAAGGTGGTGCATATGCAAGGTCCATAC |
| HW2546 | GAGCTCGGTACCCGGGGATCCAAAATATATCTGGAAACTTACCAA |
| HW2547 | GACGTCGACTCTAGAGGGATCCATTTACTCCTCCATTACCTAAGATTTG |
| HW2550 | CGCTCCATGGAGATCTCGATATGAGTAATGTTACAGCAGAACAAAAAA |
| HW2551 | TGCATGTCTGCAGGCCTCGAGTTAGTGGTGATGGTGATGATGGCATACAGCTCCACCTG |
| HW2564 | CATCAAGTTTAGTCCAACTGTCAAAATCCTCA |
| HW2611 | CGCTTAAGACGCTTTGGCTATACA |
| HW2602 | AATAACAGGTGGAACTGGATCAAGG |
| HW2603 | CTTCGTGTATTTCAGCAGCAAGTG |

# **Supplementary References**

[a] J.T. Heap, O.J. Pennington, S.T. Cartman, N.P. Minton, A modular system for *Clostridium* shuttle plasmids, J. Microbiol. Methods. 78 (2009) 79–85. doi:10.1016/j.mimet.2009.05.004.

Table S3 Genes used for MEME analysis

| ID | Fold Change | Down/Up | Protien | Site |
| --- | --- | --- | --- | --- |
| *CD17960* | 3.69 | Down | 4Fe-4S-binding domain protein | -351 |
| *CD23380* | 2.25 | Down | 4-hydroxybutyrate dehydrogenase | -356 |
| *CD23800* | 2.09 | Down | indolepyruvate oxidoreductase subunit beta | -357 |
| *CD36051* | 2.42 | Down | ferredoxin | -773 |
| *CD10550* | 2.21 | Down | electron transfer flavoprotein subunit beta/FixAfamily protein | -789 |
| *CD23410* | 2.38 | Down | 4-hydroxybutyryl-CoA dehydratase/vinylacetyl-CoA-Delta-isomerase | -928 |
| *CD23480* | 2.08 | Down | glycine/betaine reductase C | -987 |
| *CD23550* | 4.50 | Down | thiol reductase thioredoxin | -1005 |

**Table S4** Determination of the Minimum Inhibitory Concentration (MIC) for *C. difficile* strains

| Antibiotics | Minimum Inhibitory Concentration (MIC) values | | | CLSI (M11Ed9E) resistance breakpoints (µg/mL) | | |
| --- | --- | --- | --- | --- | --- | --- |
|  | WT | ∆*xtr* | ::*xtr* | S | I | R |
| Metronidazole | 16 | 8 | 16 | 8**≤** | 16 | **≥32** |
| Vancomycin | 4 | 2 | 4 | 0.5**≤** | 1 | **≥2** |

Note： S: Susceptible, I: Intermediate, R: Resistant.

**Table S5** Top 10 up-regulated genes in the Δ*xtr* strain

| **ID** | **Basemean**  **(CD630)** | **Basemean**  **(∆*xtr*)** | **FoldChange** | **log_2_FoldChange** | **Pval** | **Function** |
| --- | --- | --- | --- | --- | --- | --- |
| CD630_02940 | 9.137895177 | 244.766446 | 26.78586713 | 4.743400095 | 6.40222E-57 | ABC transporter permease (ABC) |
| CD630_08770 | 322.4399589 | 7428.215178 | 23.03751435 | 4.525913159 | 8.17996E-10 | ATP-binding cassette domain-containing protein (ABC protein) |
| CD630_08890 | 48.6657251 | 1066.526656 | 21.91535528 | 4.453870162 | 0.017795083 | adenosylmethionine decarboxylase (AdoMetDC) |
| CD630_08880 | 105.9850733 | 2259.795408 | 21.3218271 | 4.414259165 | 0.036373203 | aminotransferase class V-fold PLP-dependent enzyme (PLPDE-V) |
| CD630_22140 | 25.4798404 | 458.0044324 | 17.97516881 | 4.167933414 | 2.05787E-48 | helix-turn-helix domain-containing protein (HTH protein) |
| CD630_08740 | 758.5570834 | 12544.85457 | 16.5377858 | 4.047694184 | 1.81987E-06 | ATP-binding cassette domain-containing protein (ABC protein) |
| CD630_02920 | 78.46955364 | 1253.848524 | 15.97879007 | 3.998086265 | 3.4359E-114 | helix-turn-helix domain-containing protein (HTH protein) |
| CD630_02930 | 45.24716129 | 716.3048165 | 15.83093383 | 3.984674454 | 1.12348E-90 | ABC-type transport system, bacitracin / multidrug-family ATP-binding protein (ABC transporter) |
| CD630_10921 | 2.117171781 | 30.7845834 | 14.54042779 | 3.86199781 | 8.49292E-09 | helix-turn-helix domain-containing protein (HTH protein) |
| CD630_06180 | 9.450401452 | 118.493693 | 12.53848248 | 3.648290846 | 1.68414E-10 | LytTR family transcriptional regulator (LytTR) |

**Table S6 Top 10** down-regulated genes in the Δ*xtr* strain

| **ID** | **Basemean**  **(CD630)** | **Basemean**  **(Δ*xtr*)** | **FoldChange** | **log_2_FoldChange** | **Pval** | **Function** |
| --- | --- | --- | --- | --- | --- | --- |
| CD630_14780 | 1355.67626 | 51.37038301 | 0.03789281 | -4.721932059 | 0.006534349 | ferrous iron transport protein A (FeoA) |
| CD630_09880 | 20.89130412 | 0.909122644 | 0.043516797 | -4.522283812 | 6.40222E-57 | hypothetical protein |
| CD630_15580 | 425.2293186 | 18.96750112 | 0.044605347 | -4.486639543 | 8.17996E-10 | homoserine dehydrogenase (HSD) |
| CD630_14850 | 639.7921455 | 28.66050693 | 0.044796591 | -4.480467249 | 0.017795083 | DUF3793 family protein (DUF3793) |
| CD630_15810 | 278.0948074 | 14.89524667 | 0.053561758 | -4.222652886 | 0.036373203 | exosporium morphogenetic protein CdeM (CdeM) |
| CD630_32830 | 1850.554114 | 105.3653377 | 0.056937183 | -4.134485081 | 2.05787E-48 | glycyl-radical enzyme activating protein (GRAE) |
| CD630_32820 | 11819.16288 | 722.292809 | 0.061112011 | -4.032400239 | 1.81987E-06 | glycyl radical protein (GRP) |
| CD630_30120 | 3926.28584 | 260.3828391 | 0.066317851 | -3.914458933 | 3.4359E-114 | alpha-mannosidase (α-Man) |
| CD630_10870 | 942.2061759 | 64.78243954 | 0.068756119 | -3.862368086 | 1.12348E-90 | zinc transporter ZupT (ZupT) |
| CD630_17950 | 22.11577936 | 1.615422916 | 0.073043906 | -3.775092269 | 8.49292E-09 | DUF1883 domain-containing protein (DUF1883) |

**Table S7** Determination of the Minimum Inhibitory Concentration (MIC) for *C. difficile* strains

| Antibiotics | Minimum Inhibitory Concentration (MIC) values | | | CLSI (M11Ed9E) resistance breakpoints (µg/mL) | | |
| --- | --- | --- | --- | --- | --- | --- |
|  | WT | ∆*xtr* | Δ*xtr*::*4Fe-4S_BP* | S | I | R |
| Metronidazole | 16 | 8 | 16 | 8**≤** | 16 | **≥32** |
| Vancomycin | 4 | 2 | 4 | 0.5**≤** | 1 | **≥2** |

Note： S: Susceptible, I: Intermediate, R: Resistant.
