# Supplementary material for: Xtr-mediated regulation of 4Fe-4S binding protein gene confers resistance to oxidative and antibiotic stress in Clostridioides difficile
Source: Front Microbiol. 2026 May 29;17:1817884. doi: 10.3389/fmicb.2026.1817884 (PMC13260099; doi:10.3389/fmicb.2026.1817884)
Supplement: Supplementary file 2 [file Supplementary_file_2.docx]

Supplementary Material

Supplementary Figures


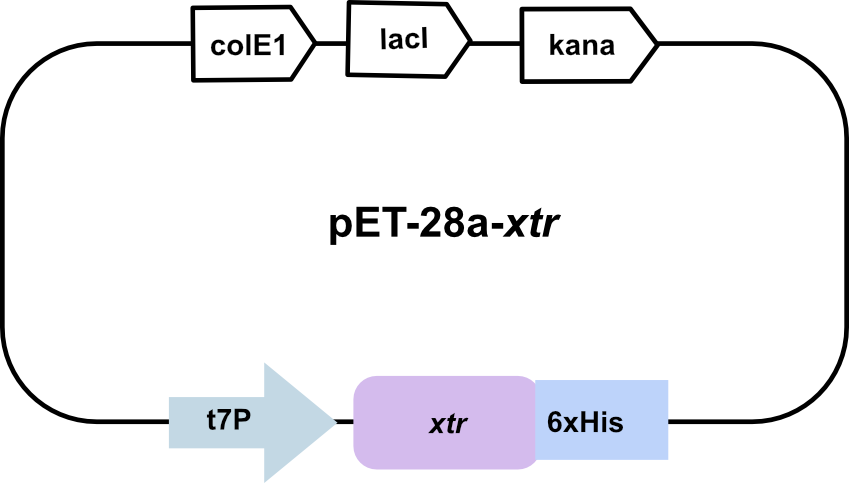


Figure S1. Schematic map of the recombinant expression vector pET-28a-*xtr*

This vector is based on the pET-28a backbone and contains the colE1, lacI, and kanamycin resistance gene (kana) for plasmid replication and selection. The *xtr* gene was cloned downstream of the T7 promoter and fused with a 6 × His tag at the C-terminus to facilitate protein purification. This vector allows for inducible expression of the Xtr-His fusion protein under the control of a T7 RNA polymerase-driven transcription system.


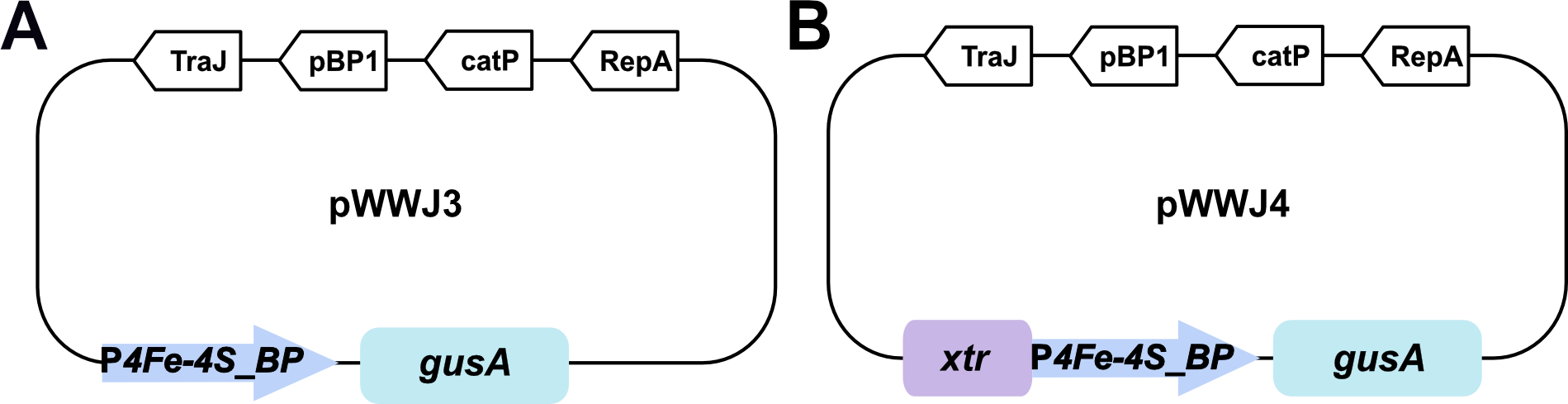


Figure S2. Schematic map of the recombinant expression vectors pWWJ3 and pWWJ4 carrying the *gusA* reporter gene

(A) Structure of plasmid pWWJ3: Based on an autonomous replication vector, it contains TraJ, pBP1, catP, and RepA. The reporter gene *gusA* (β-glucuronidase) is driven by the *4Fe-4S_BP* promoter. (B) Structure of plasmid pWWJ4: Built upon pWWJ3, the *xtr* gene was inserted upstream of the *4Fe-4S_BP* promoter to analyze the regulatory effect of *xtr* on *gusA* expression.


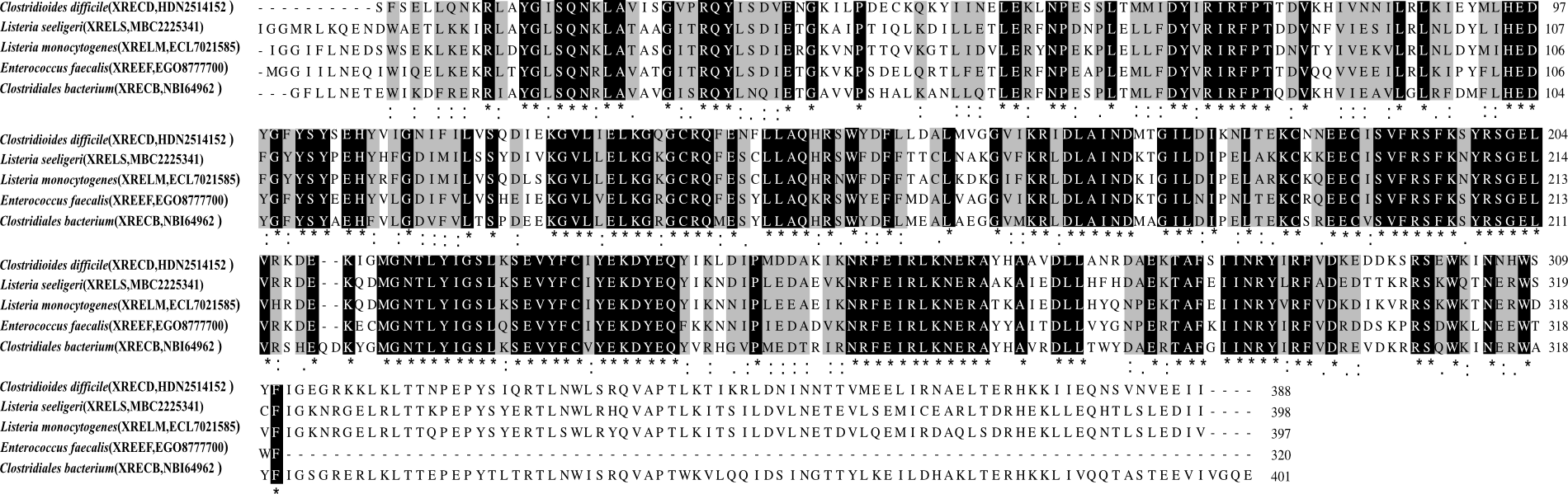
Figure S3. Multiple Sequence Alignment Analysis

This figure presents the results of an amino acid sequence alignment of XRE family proteins from five different bacteria (*C. difficile*, *Listeria seeligeri,* *Listeria monocytogenes*, *Enterococcus faecalis*, *Clostridiales bacterium*). Protein database accession numbers (e.g., XRECD, HDN2514152) are indicated to the right of each sequence. Symbols below the sequences indicate conservation levels: "*" denotes identical amino acids across all species, ":" denotes highly similar amino acids, and "." denotes partially similar amino acids.


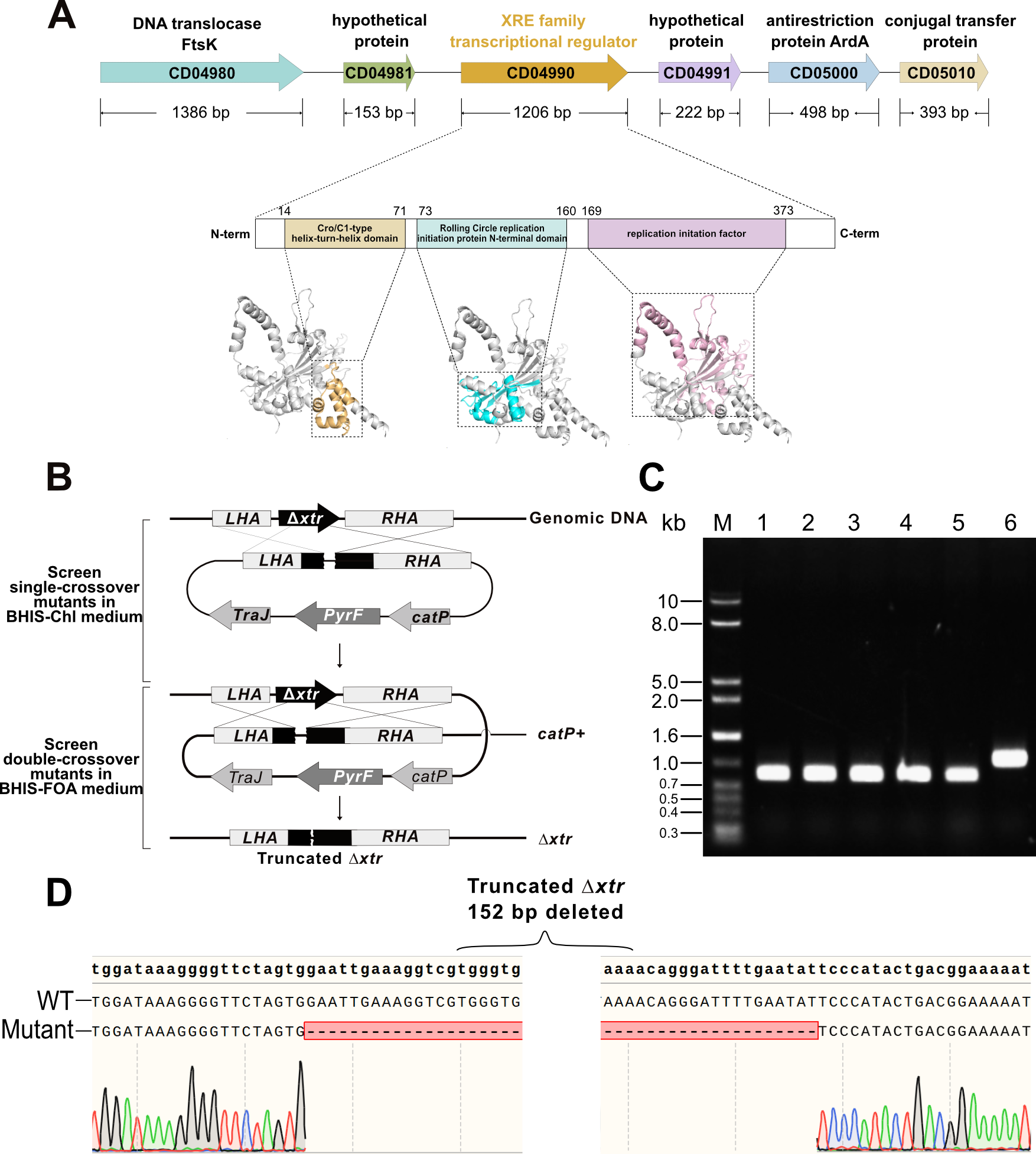


Figure S4. Gene context of the *xtr* gene and ACE mediated gene deletion of the *xtr*

(A) Gene context and functional domain annotations. The top panel shows the gene cluster (including DNA transposase, hypothetical protein, XRE family transcriptional regulator, anti-restriction protein, and conjugation transfer protein) and the length of each gene. The bottom panel shows predicted functional domains, including the N-terminal Helix-turn-helix (HTH) domain, the intermediate Raring-cyclo cyclase domain, and the C-terminal replication factor domain, along with predicted 3D protein models. (B) Schematic representation of *xtr* knockout. The *xtr* gene was knocked out using ACE homologous recombination. Starting with a Δ*pyrF* background strain, single crossover events were selected on BHI agar. Subsequent double crossover events were identified through screening on BHI plates containing 5-fluoroorotic acid (BHIS-FOA), resulting in the *xtr* knockout strain (Δ*xtr*). (C) PCR verification of the Δ*xtr* knockout strain. (D) Sequencing verification of the Δ*xtr* knockout strain.


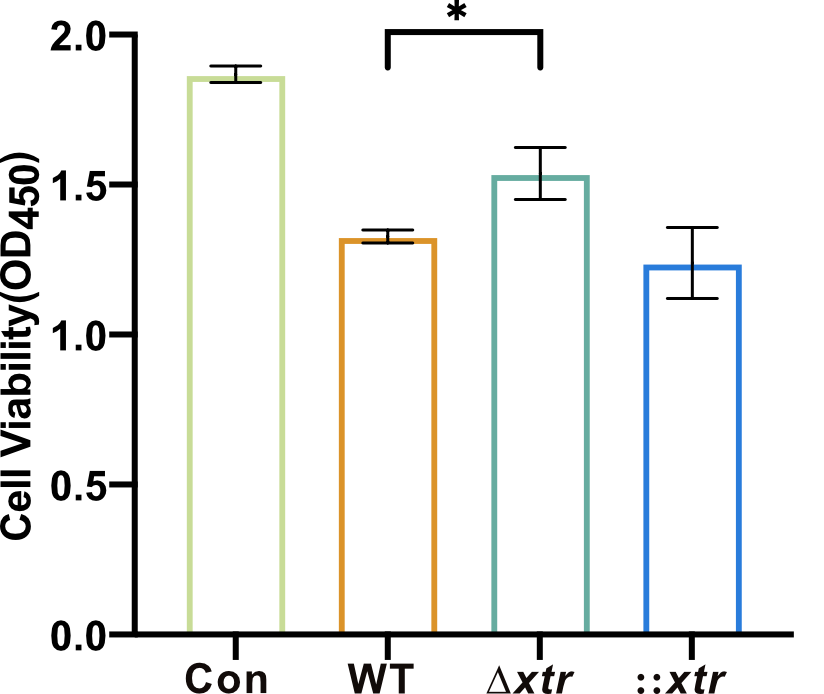


Figure S5. Effect of ∆*xtr* on *C. difficile*-induced cytotoxicity in Vero cells

Vero cells were incubated with filtered culture supernatants from the indicated *C. difficile* strains (Con: control, WT, Δ*xtr*, ::*xtr*) for 24 h. Cell viability was measured by CCK-8 assay and expressed as OD_450_ values. Data are mean ± SEM (n=3). * *P* < 0.05.


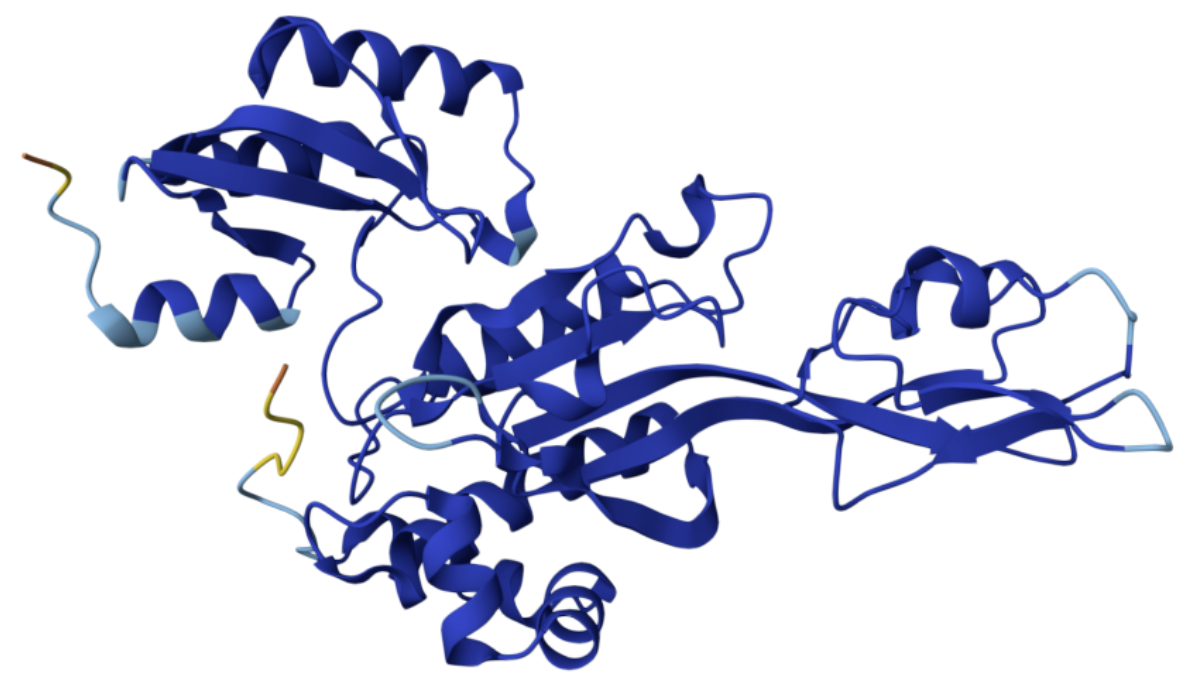


Figure S6. The predicted three-dimensional structure of the 4Fe-4S binding protein

The structure was predicted by AlphaFold 2 with an overall pLDDT score of 94.38, indicating extremely high confidence. The protein adopts a characteristic β-barrel core topology, with a rigid scaffold formed by multiple parallel and antiparallel β-sheets (dark blue) stabilized by α-helices and flexible loop regions (light blue/cyan). A well-defined, structurally stable binding pocket for 4Fe-4S cluster coordination is located in the core region of the protein, providing a structural basis for the iron-sulfur cluster binding and electron transfer function of this protein. The N- and C-termini are colored in red and orange, respectively.


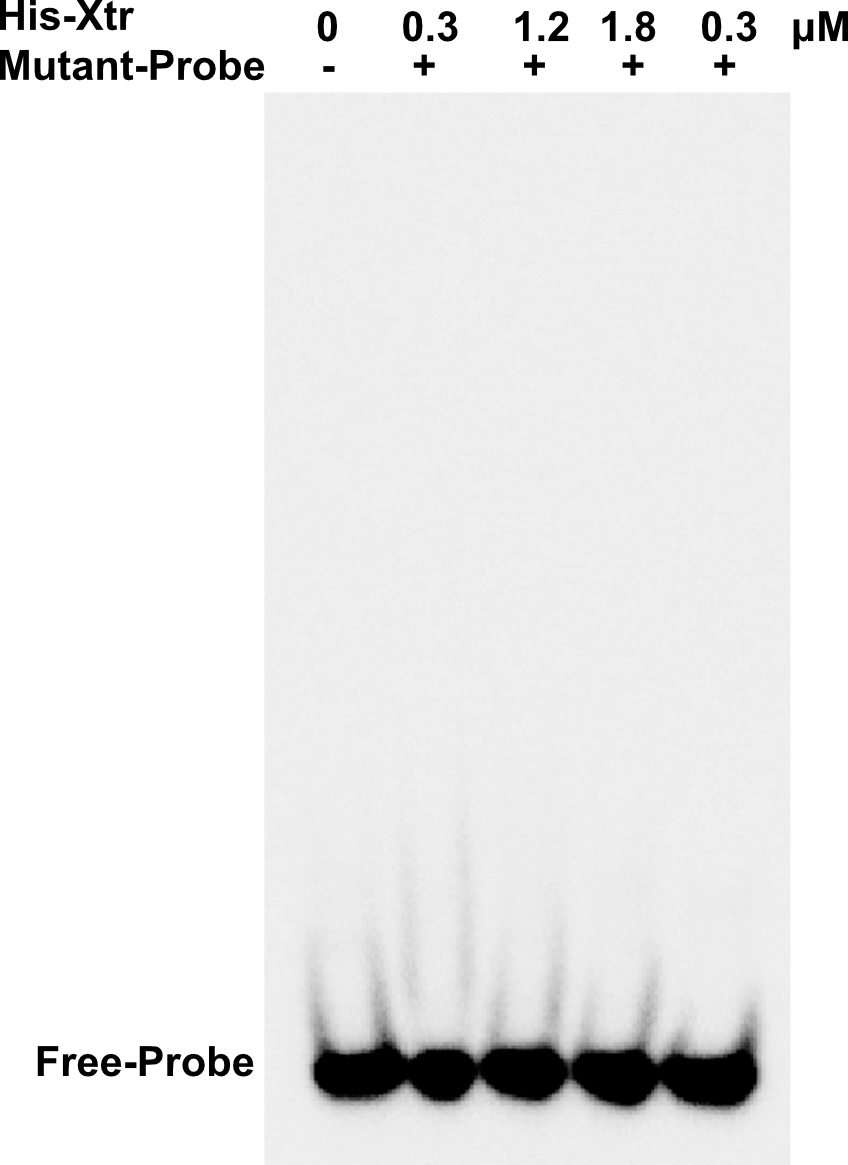


Figure S7. Electrophoretic mobility shift analysis of His-Xtr protein with the mutant probe

The upper horizontal axis indicates the concentration gradient of His-Xtr protein, which is 0, 0.3, 1.2, 1.8, and 0.3 μM, respectively. Except for the 0 μM lane, all other lanes contain an equal amount of the mutant probe (labeled as "+"). The 0 μM lane serves as a negative control containing only the free probe (labeled as "-"). The bottom band is labeled "Free-Probe," indicating the unbound mutant probe not associated with the prote.


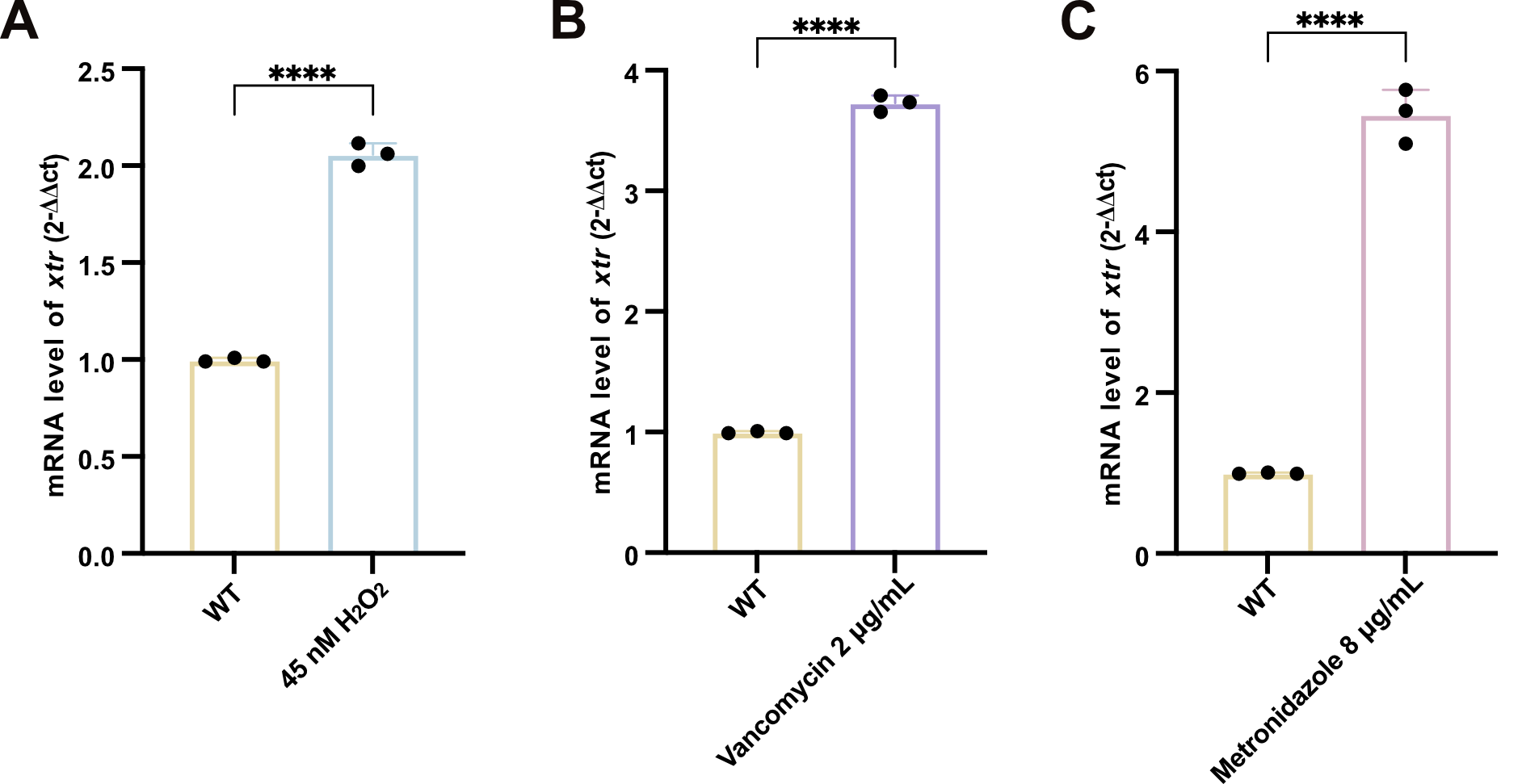


Figure S8. *xtr* is significantly upregulated in *C. difficile* in response to oxidative stress and antibiotics

*xtr* mRNA levels were determined by qRT‑PCR in WT treated with 45 nM H_2_O_2_ (A), 2 μg/mL vancomycin (B), and 8 μg/mL metronidazole (C), with untreated WT as the control. Data are mean ± SEM (n=3).**** *P* < 0.000.
